# Efficacy of Er:YAG laser in removal of impacted mandibular third molars (a randomized controlled clinical trial)

**DOI:** 10.1186/s12903-026-08790-w

**Published:** 2026-06-05

**Authors:** Karim A. Elhaw, Ahmed A. Abdelhakim, Arnabat. Dominguez J, Riham M. Eldibany, España. Tost A

**Affiliations:** 1 Oral Laser Applications Department, Faculty of Dentistry, Alexandria University, Alexandria, Egypt; 2 Prosthodontics Department, Faculty of Dentistry, Alexandria University, Alexandria, Egypt; 3 Oral Surgery, Faculty of Dentistry, Barcelona University, Barcelona, Spain; 4 Oral and Maxillofacial Surgery, Faculty of Dentistry, Alexandria University, Alexandria, Egypt; 5 Oral Surgery and Implantology, University of Barcelona Dental School, Researcher of the IDIBELL Institute, Barcelona, Spain

**Keywords:** Er:YAG laser, Impacted mandibular third molars, Bone removal, Postoperative complications, Bone healing

## Abstract

**Background:**

Removal of impacted mandibular third molars is associated with postoperative discomfort and delayed bone healing. Er:YAG laser osteotomy has been proposed as a minimally invasive alternative to conventional rotary instrumentation.

**Aim:**

To compare Er:YAG laser osteotomy and conventional rotary osteotomy regarding postoperative radiographic bone density and clinical healing outcomes following impacted mandibular third molar removal (pain, edema, and trismus).

**Methods:**

Twenty-four patients (*n* = 24) were randomized equally (laser *n* = 12; rotary *n* = 12). CBCT scans were obtained preoperatively and at 3 months for voxel-based grayscale bone density assessment. Standardized 3D ellipsoid VOIs were used. A single trained examiner performed all CBCT measurements twice, two weeks apart (ICC > 0.90). Postoperative pain, edema, and trismus were clinically assessed.

**Results:**

Immediate postoperative bone density showed no significant difference between groups (*p* = 0.319). At 3 months, bone density was significantly higher in the laser group compared with the rotary group (median: 310.7 vs. 270.0; *p* = 0.017), with a moderate effect size (*r* = 0.477). Bone density significantly increased over time in both groups (*p* = 0.002), with a significantly greater increase observed in the laser group (*p* = 0.017). Pain scores on day 2 were significantly lower in the laser group (*p* = 0.020). Edema and trismus showed significant improvement over time within both groups, with no statistically significant differences between groups. Operation time was significantly longer in the laser group.

**Conclusion:**

Er:YAG laser osteotomy was associated with lower postoperative pain and higher radiographic grayscale values at 3 months compared with rotary osteotomy. Edema and trismus improved over time in both groups, with no significant differences between the two techniques.

**Clinical relevance:**

Er:YAG laser may improve patient comfort by reducing postoperative pain and may be associated with improved radiographic bone healing. However, further studies are needed to confirm its long-term effects on bone density.

**Trial registration:**

ClinicalTrials.gov (Identifier: NCT07297043). Registered 09 December 2025 (retrospective).

**Supplementary Information:**

The online version contains supplementary material available at 10.1186/s12903-026-08790-w.

## Background

Impacted mandibular third molar removal is one of the most frequently performed procedures in oral and maxillofacial surgery [1]. Conventional rotary instruments are effective for bone cutting; however, their use is associated with mechanical vibration and thermal stress, which may negatively influence postoperative comfort and bone healing [2].

Er:YAG laser osteotomy provides micro-explosive ablation with minimal thermal damage to surrounding tissues [3]. This technique has been associated with reduced intraoperative bleeding, improved patient comfort, and potentially enhanced postoperative healing. Nevertheless, high-quality clinical trials directly comparing postoperative bone density following laser versus rotary osteotomy remain limited [4].

Therefore, this study aims to compare Er:YAG laser osteotomy and conventional rotary osteotomy regarding postoperative bone density and clinical healing parameters, including pain, edema, and trismus [5].

An impacted tooth is one that fails to emerge into the dental arch within the expected time frame. This condition arises when adjacent teeth, dense overlying bone, excessive soft tissue, or genetic abnormalities hinder the eruption process [6]. The third molars, also known as wisdom teeth, are the most frequently impacted teeth because they are the last to emerge. As a result, they often face inadequate space for proper eruption [7]. Impacted third molars are not only a common dental concern but also a significant challenge in oral and maxillofacial surgery. These teeth are prone to causing several complications, including pain, infection, and damage to adjacent teeth, necessitating their removal in most cases [8].

The decision to extract impacted third molars involves evaluating the potential benefits and risks. In many cases, surgical bone removal is required to access the impacted teeth [9]. This is a routine outpatient procedure, performed worldwide in oral and maxillofacial surgery practices [10]. However, despite its commonality, this procedure is associated with post-operative complications such as pain, swelling, trismus, and infection [11]. There is still no clear consensus on the optimal method for minimizing these complications, which makes the surgical technique and tools used critical factors in improving patient outcomes [12].

The traditional method of extracting impacted third molars often involves the use of rotary tools, which can create several challenges. These include bone debris accumulation, overheating of surrounding bone, and patient discomfort. While rotary systems have been refined over the years to address some of these issues, they remain less than ideal [13].

Laser dentistry has revolutionized the way oral and maxillofacial surgeries are performed. The Er:YAG laser is highly effective in removing both hard and soft tissues with minimal heat damage [14]. This laser’s ability to target the water and hydroxyapatite components of bone, while penetrating only 0.1 mm of hard tissue, allows for precise and controlled removal with significantly reduced thermal impact [15]. The use of this laser not only minimizes bone temperature but also offers a clean surgical field, which is an essential factor in enhancing recovery and reducing post-operative complications [16].

One of the primary advantages of using the Er:YAG laser is its bactericidal effect. The laser’s energy helps sterilize the surgical site, reducing the risk of infection [17]. Additionally, the laser’s biostimulating effects seem to accelerate the healing process by enhancing cellular activity and tissue regeneration [18]. These characteristics make the Er:YAG laser a promising tool for improving surgical outcomes and reducing recovery times in dental procedures [19].

This study aims to compare the effectiveness of the Er:YAG laser with traditional rotary drilling systems in the removal of impacted mandibular third molars.

## Materials and methods

### Study design

**Fig. 1:**
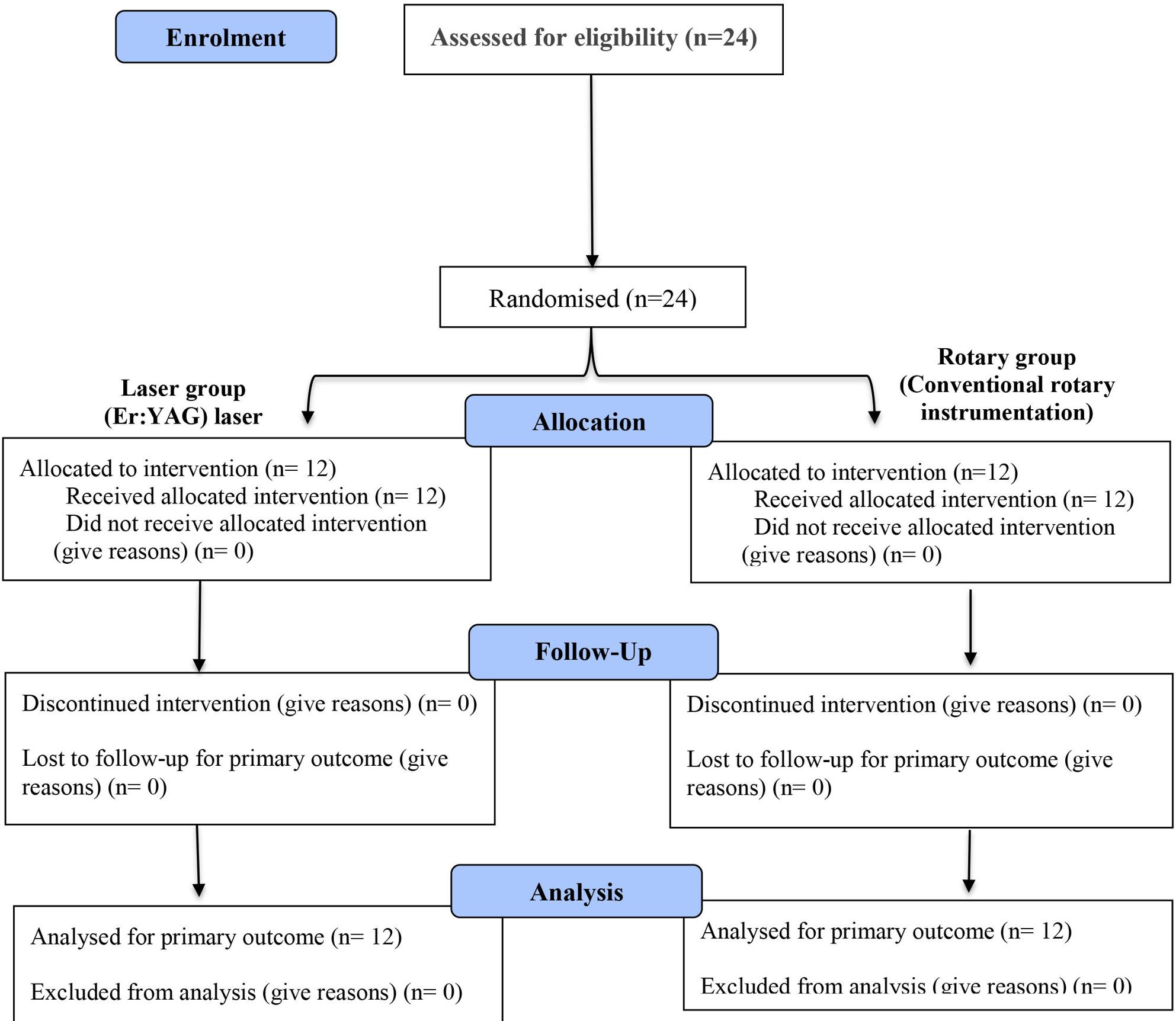
CONSORT 2010 Flow Diagram

This prospective randomized controlled clinical trial (*n* = 24) was conducted at the Dental Laser Unit, Faculty of Dentistry, Alexandria University. Recruitment occurred from 14 January 2024 to 27 January 2025. Ethical approval was obtained prior to recruitment (0969-09/2024-IORG 0008839). Trial registration was completed retrospectively on 09 December 2025. Written informed consent was obtained from all participants prior to inclusion in the study. This randomized clinical trial was designed, conducted, and reported in accordance with the CONSORT (Consolidated Standards of Reporting Trials) 2010 statement. A completed CONSORT checklist and flow diagram are provided (Fig. 1). The CONSORT checklist is available in Supplementary Table S1, and the predefined primary and secondary outcomes are detailed in Supplementary Table S2.

This trial was registered at ClinicalTrials.gov (NCT07297043) on 09 December 2025. Although the trial registration was completed retrospectively due to administrative delay, the study protocol and outcomes were predefined prior to participant recruitment, and no modifications were made during the study conduct. The full pre-specified study protocol is available in the Additional file.

### Laser parameters

Er:YAG (Fotona), H02 handpiece, wavelength 2940 nm, QSP mode, SSP pulse modality, pulse energy 350 mJ, frequency 10 Hz, power 3.5 W, water level 3, air level 6, non-contact technique. Spot diameter was 1.3 mm (radius = 0.65 mm). Energy density was approximately 26.5 J/cm², and power density was approximately 265.2 W/cm².

### CBCT radiographic assessment

Standardized CBCT scans were acquired preoperatively and at 3 months using identical imaging parameters. Bone density was measured using 3D ellipsoid VOIs (radii 6.12 mm, 6.05 mm, 2.23 mm; volume ≈ 2.23 cc). CBCT analysis was performed using OnDemand3D App software (version 1.0; CyberMed, Seoul, South Korea). Scans were acquired using a J. Morita R100 CBCT unit (J. Morita Mfg. Corp., Kyoto, Japan) with a voxel size of 0.125 mm, field of view of 80 × 80 mm, tube voltage of 90 kV, tube current of 8 mA, and an exposure time of approximately 9.4 s. A standardized ellipsoid volume of interest (VOI) was positioned at the center of the extraction socket using reproducible anatomical landmarks. Measurements were performed twice by the same blinded examiner with a two-week interval. Intra-observer reliability was excellent (ICC = 0.93, 95% CI: 0.88–0.97).

The examiner responsible for CBCT analysis was blinded to the clinical outcome data and patient allocation during image assessment. All scans were anonymized and coded before VOI placement and grayscale analysis.

### Sample size calculation

Sample size was estimated assuming a 95% confidence level and 80% study power. Based on a previous study, the mean mouth opening after 7 days was 33.45 ± 2.32 mm for the conventional extraction technique and 30.85 ± 1.96 mm for the laser group [20]. Based on the difference between two independent means using a two-sided independent samples t-test, a minimum sample size of 12 patients per group was required, corresponding to an effect size of 1.211. Therefore, the total sample size was 24 patients (12 patients per group).

Sample size calculation was performed according to Rosner’s [21] method using G*Power software version 3.0.10 [22].

At the time of study planning, limited published data were available regarding CBCT grayscale changes after Er:YAG osteotomy; therefore, sample size estimation was based on the closest clinically relevant continuous outcome reported in previous laser-assisted third molar studies.

### Participants

**Fig. 2:**
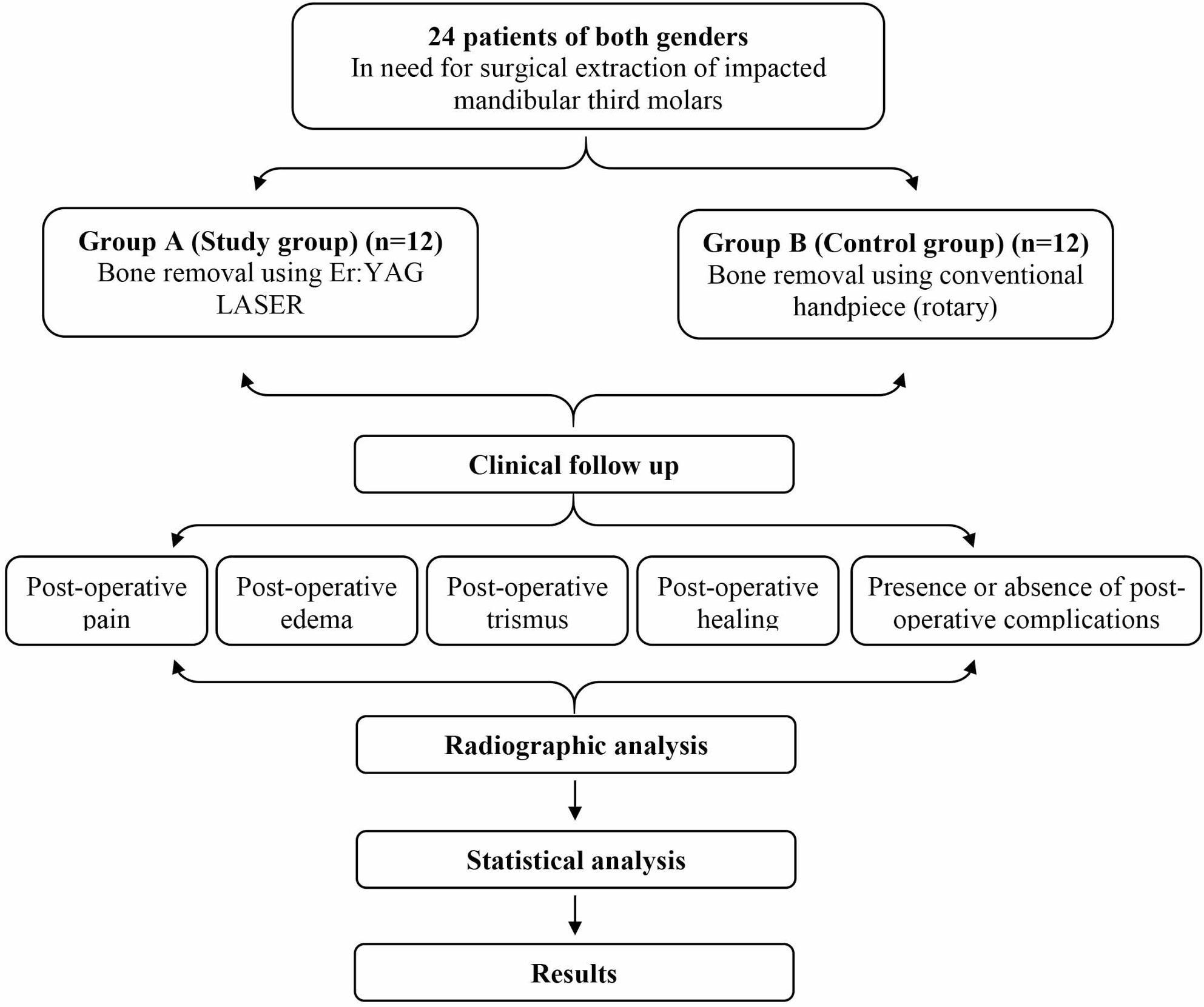
Study flow diagram

Twenty-four patients (12 males and 12 females; age range 18–45 years) with mesioangular bony impacted mandibular third molars were included. All patients were systemically healthy (ASA I or II), and none had pathologies in adjacent teeth. (Fig. 2)

### Inclusion criteria

Patients aged between 18 and 45 years presenting with mesioangular impacted mandibular third molars indicated for surgical extraction were included in the study. All participants were classified as American Society of Anesthesiologists (ASA) physical status I or II. Informed consent was obtained from each participant, confirming their willingness to comply with the scheduled follow-up visits.

### Exclusion criteria

Patients with systemic diseases classified as ASA III or higher, or any condition that could contraindicate surgery or impair wound healing were excluded. Those receiving antibiotic pre-medication, chronic NSAID therapy, or any drug affecting healing were also excluded. Patients with acute pericoronitis, severe periodontal disease, contraindications to study medications or local anesthesia, or requiring extraction of another tooth at the same surgical site were excluded. Additionally, subjects with pericoronal or periapical pathology of adjacent teeth were not included.

### Randomization and allocation

Patients were randomly assigned to one of two groups (*n* = 12 each) using a computer-generated list. Allocation concealment was achieved through opaque, sequentially numbered, sealed envelopes prepared and opened by an independent assistant not involved in treatment.

- Study group: 12 patients with mesioangular impacted mandibular third molars underwent bone removal using the Er:YAG laser.
- Control group: 12 patients with mesioangular impacted mandibular third molars underwent bone removal using a conventional rotary surgical handpiece and fissure bur.

### Preoperative preparation

**Fig. 3:**
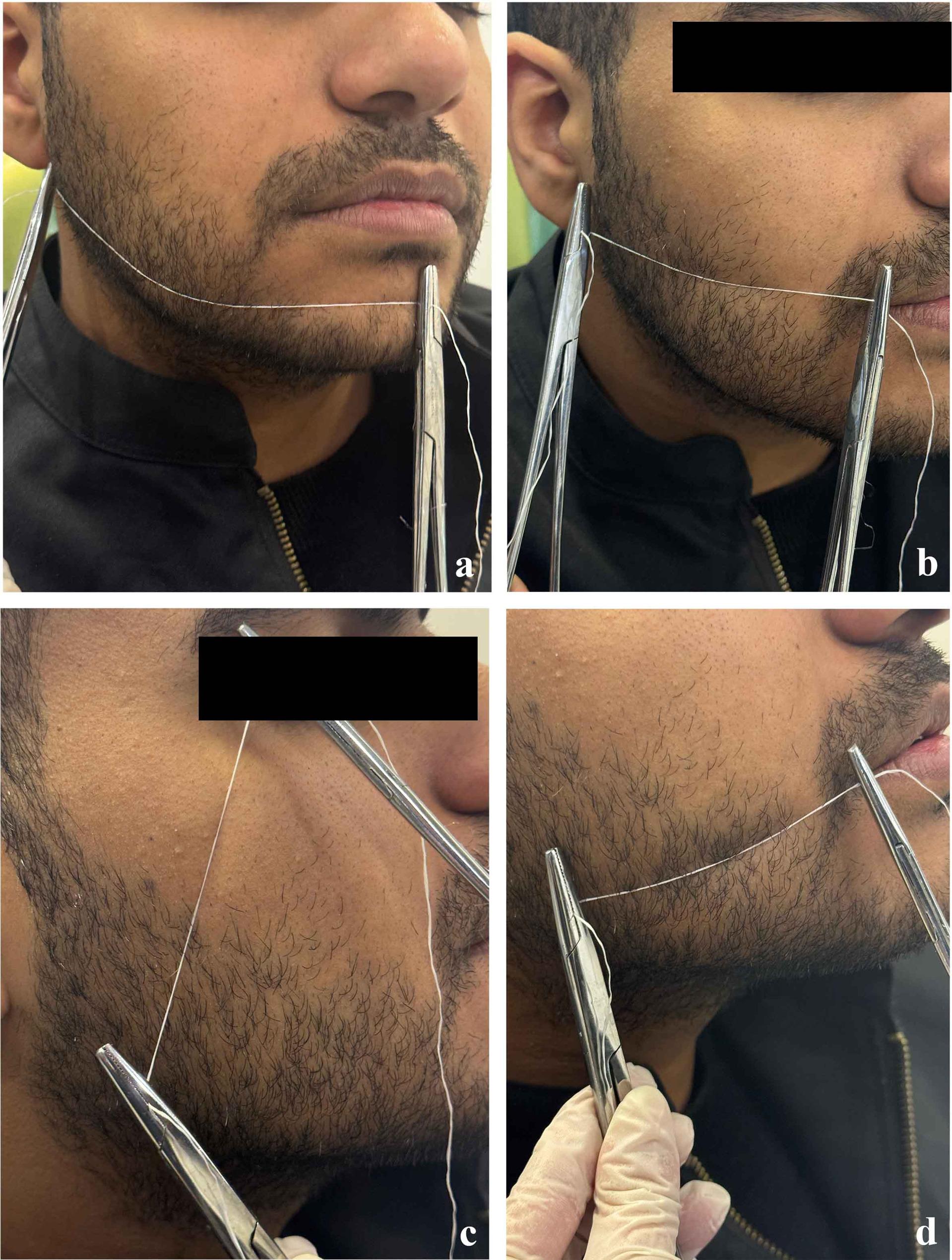
Preoperative measurements for swelling: **a**, **b** measurement from the mandibular angle to the mouth corner and **c**, **d** measurement from the ear lobule to the mouth corner

**Fig. 4:**
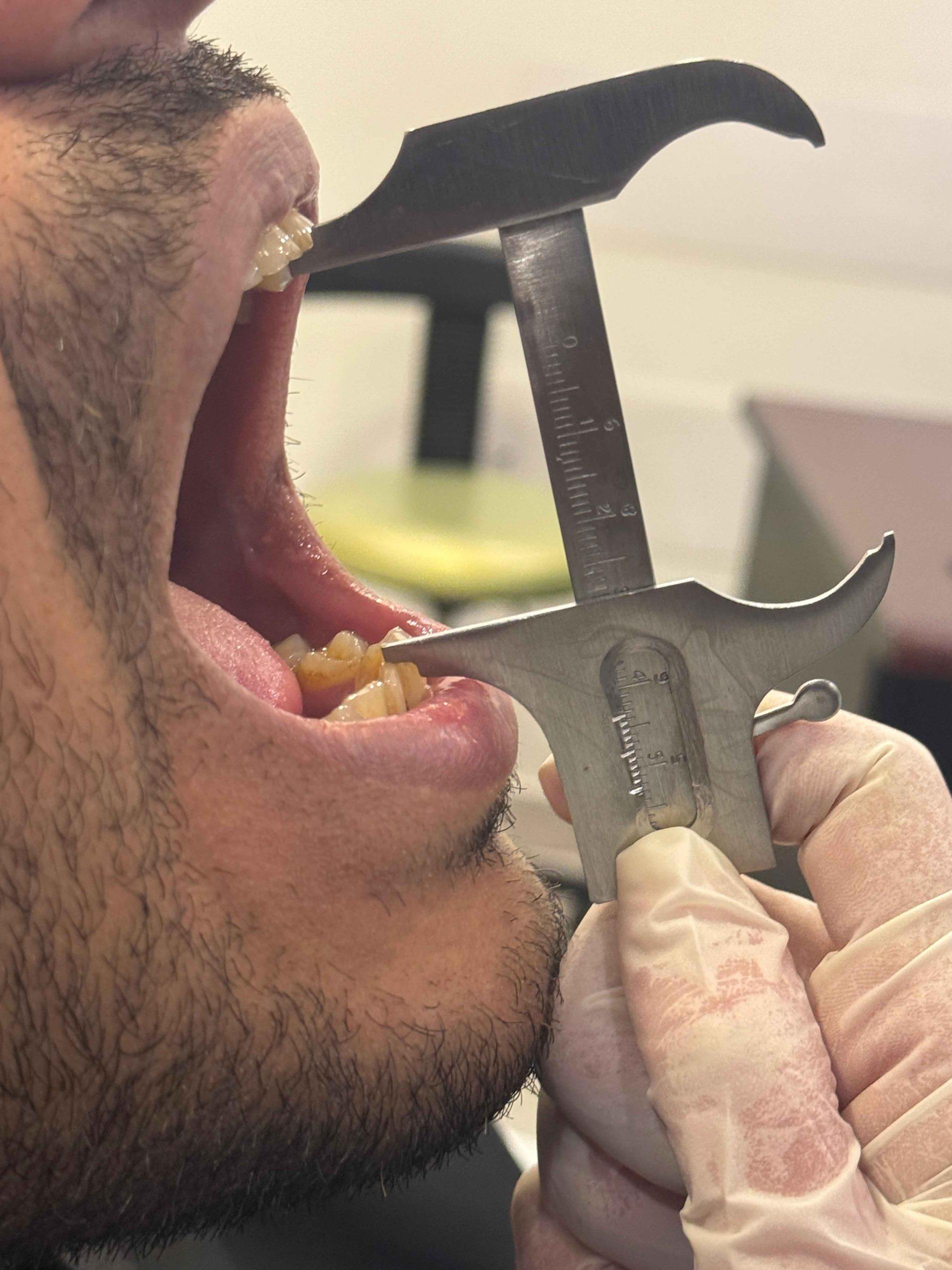
Preoperative inter-incisal opening

A standard clinical and radiographic examination was performed for all patients, including cone-beam computed tomography (CBCT) to confirm angulation and depth of impaction. All surgical procedures were carried out under local anesthesia (inferior alveolar, lingual, and long buccal nerve block with 2% lidocaine hydrochloride with 1:100,000 epinephrine (Xylocaine^®^, AstraZeneca, Egypt). (Figs. 3a-d and 4)

### Surgical procedure

#### Incision and flap design

**Fig. 5:**
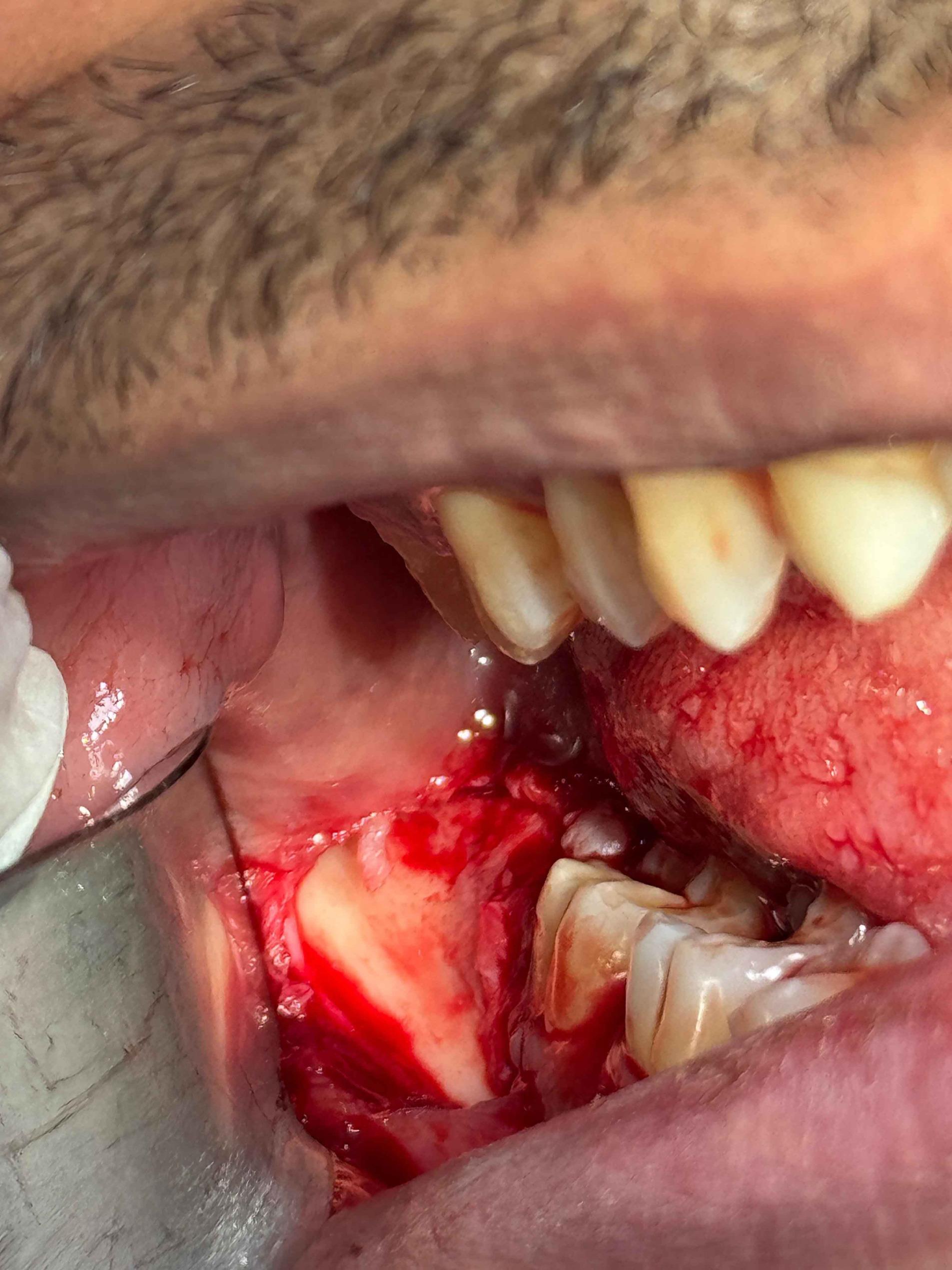
Reflected mucoperiosteal flap

In both groups, a triangular mucoperiosteal flap was performed. The incision extended buccally around the cervical margin of the mandibular second molar, continued into the interproximal space between the first and second molars, and was extended obliquely at a 45° angle toward the mucobuccal fold. The flap was elevated using a periosteal elevator to provide adequate access and visibility. (Fig. 5)

#### Study group (laser-assisted)

**Fig. 6:**
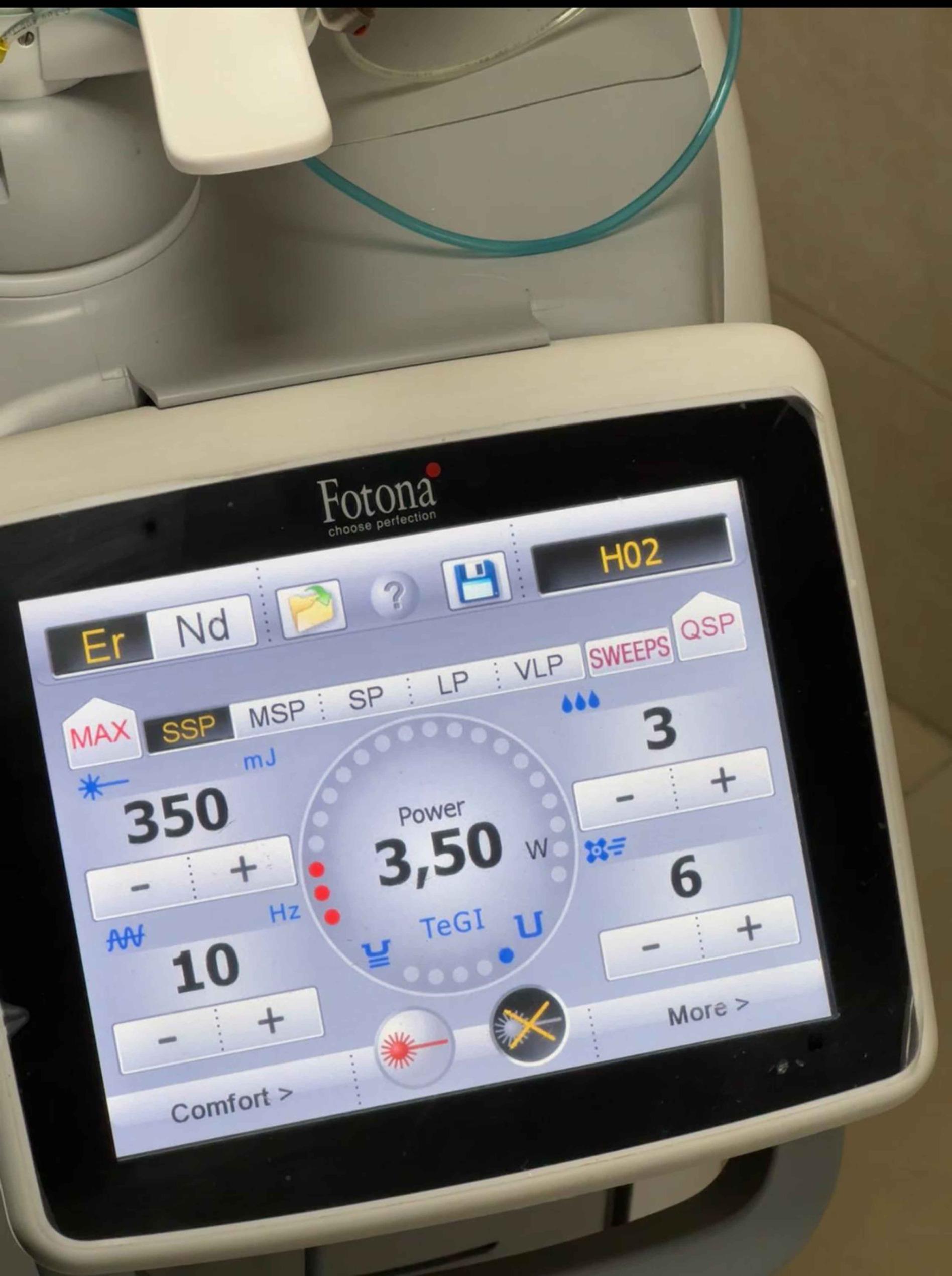
Er:YAG Laser Parameters

**Fig. 7:**
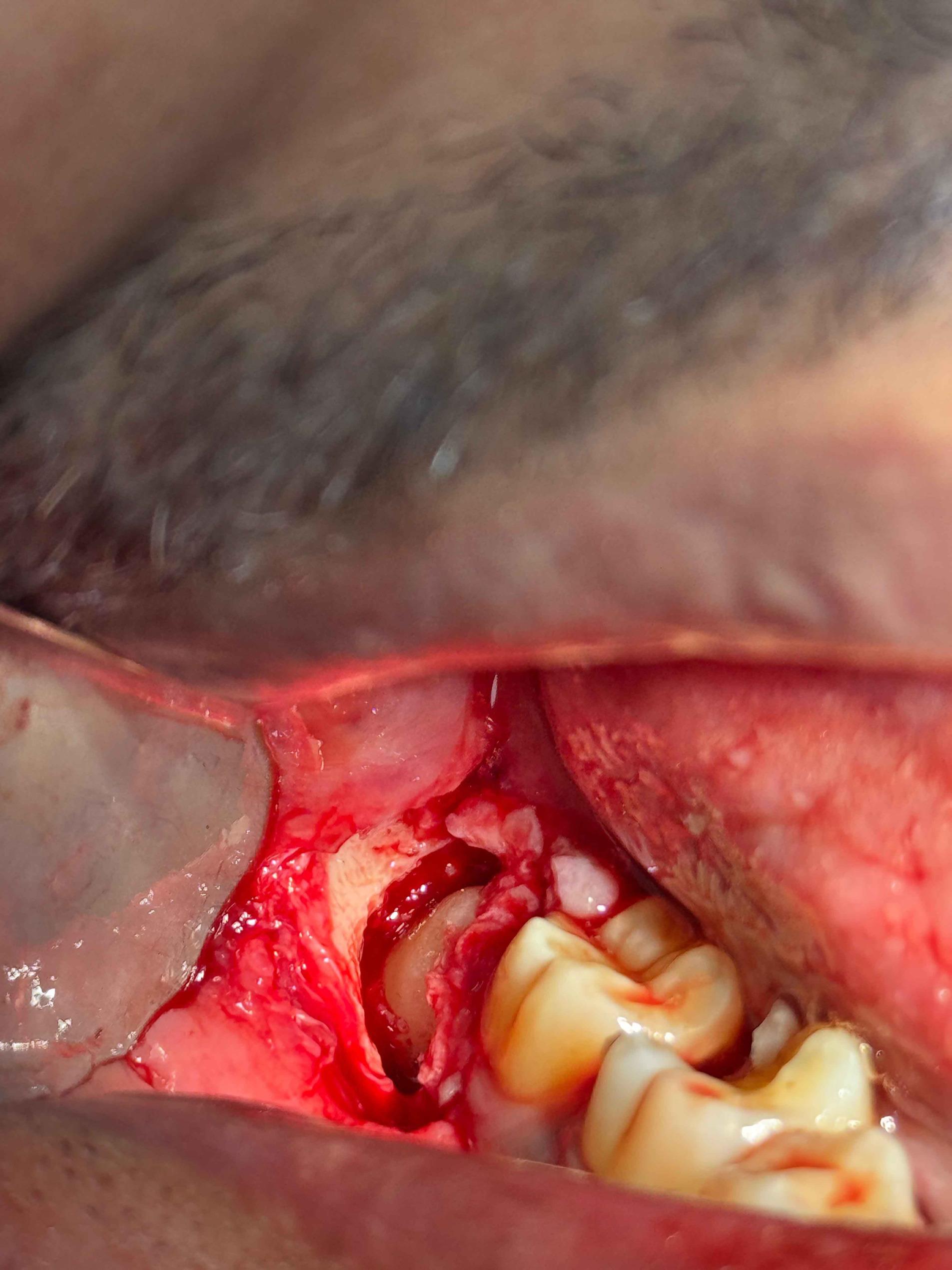
Bone guttering and tooth exposure

**Fig. 8:**
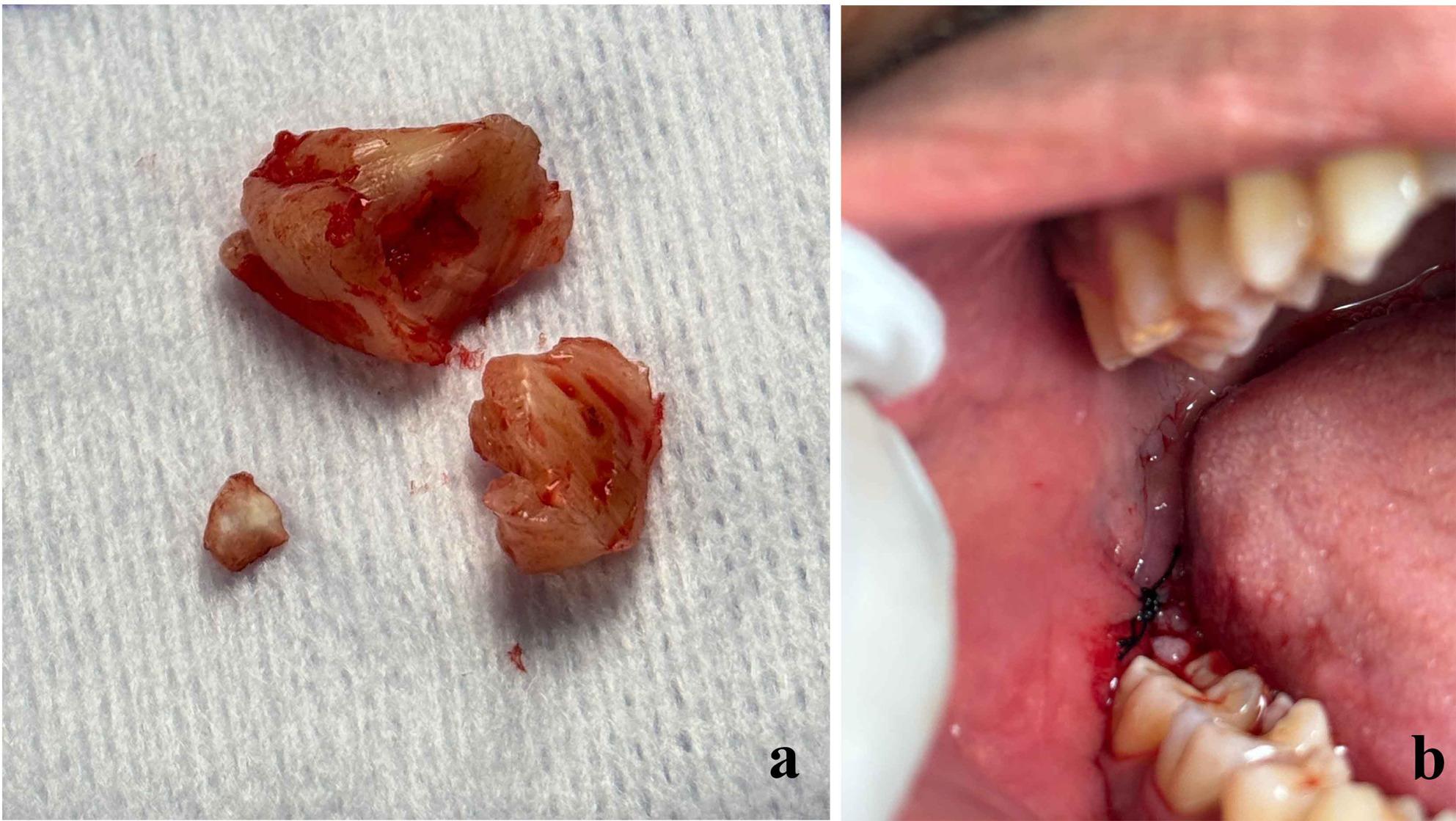
**a** Extracted tooth; **b** Sutured flap edges

In the study group, osteotomy was performed using an Er:YAG laser in a buccal and distal guttering approach. A diagonal split of the interlocked mesial cusp was created to facilitate tooth sectioning. A straight elevator was then applied at the mesial aspect of the tooth neck and rotated distally and occlusally to achieve luxation and removal. Tooth follicle remnants were removed with a curette, and irregular bony margins were refined using a bone file. The surgical site and socket were irrigated with sterile saline, and the flap was subsequently repositioned and secured using 3 − 0 black silk interrupted sutures, followed by placement of a pressure pack. (Table 1; Figs. 6, 7 and 8a, b)

**Table 1:** Er:YAGLASERParameters

Parameter	Formula / Calculation	Result		Unit
Given Data				
Power	–	3.50		W
Energy	–	350		mJ
Frequency	–	10		Hz
Diameter of the tip (2r)	–	1.3		mm
Solution steps				
Radius of the tip (r)	$\:r=\:\frac{1.3}{2}$	0.65		mm
Converted Radius	$\:\frac{0.65}{10}$	0.065		cm
Surface Area of the tip	πr^2^ = 3.14 × (0.065)^2^	0.0132		cm^2^
Energy Density	$\:\frac{E}{Area}=\:\frac{350\times{10}^{-3}}{0.0132}$	26.515	J/cm^2^	
Power Density	$\:\frac{Power}{Area}=\:\frac{3.50}{0.0132}$	265.1515	W/cm^2^	

#### Control group (rotary-assisted)

The procedure was identical, except that bone removal and sectioning were performed with a fissure surgical bur mounted on a rotary surgical handpiece.

#### Post-operative care

All patients received standardized postoperative instructions and medications:

- Antibiotics: amoxicillin/clavulanic acid (Augmentin^®^, GlaxoSmithKline, Egypt) 1 g orally, twice daily for four days.
- Analgesics: Paracetamol 500 mg orally three times daily for three days.
- Cold application: Extraoral ice packs applied intermittently for the first 6 h postoperatively.
- Diet: Soft diet recommended for the first 24 h.
- Oral hygiene: Antiseptic mouthrinse initiated on the second postoperative day and continued for 7 days.
- Suture removal: Performed at 7 days postoperatively.

### Clinical follow-up and outcome measures

#### Pain assessment

Pain was assessed using a 10-point visual analogue scale (VAS) [23] on postoperative days 2 and 7. Patients rated their pain intensity on a scale ranging from 0 (no pain) to 10 (worst imaginable pain).

#### Edema assessment

**Fig. 9:**
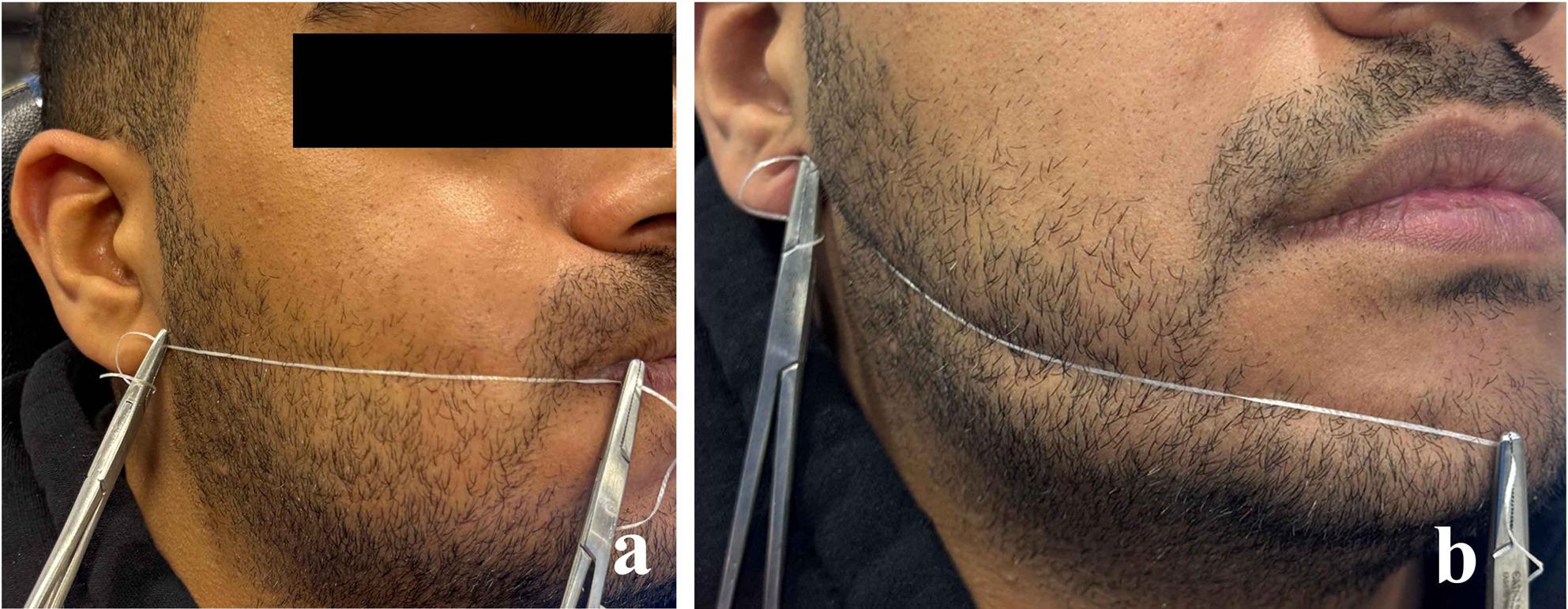
Second-day edema: **a**, **b** representative clinical photographs of edema assessment of the same patient in the laser group on the second postoperative day

**Fig. 10:**
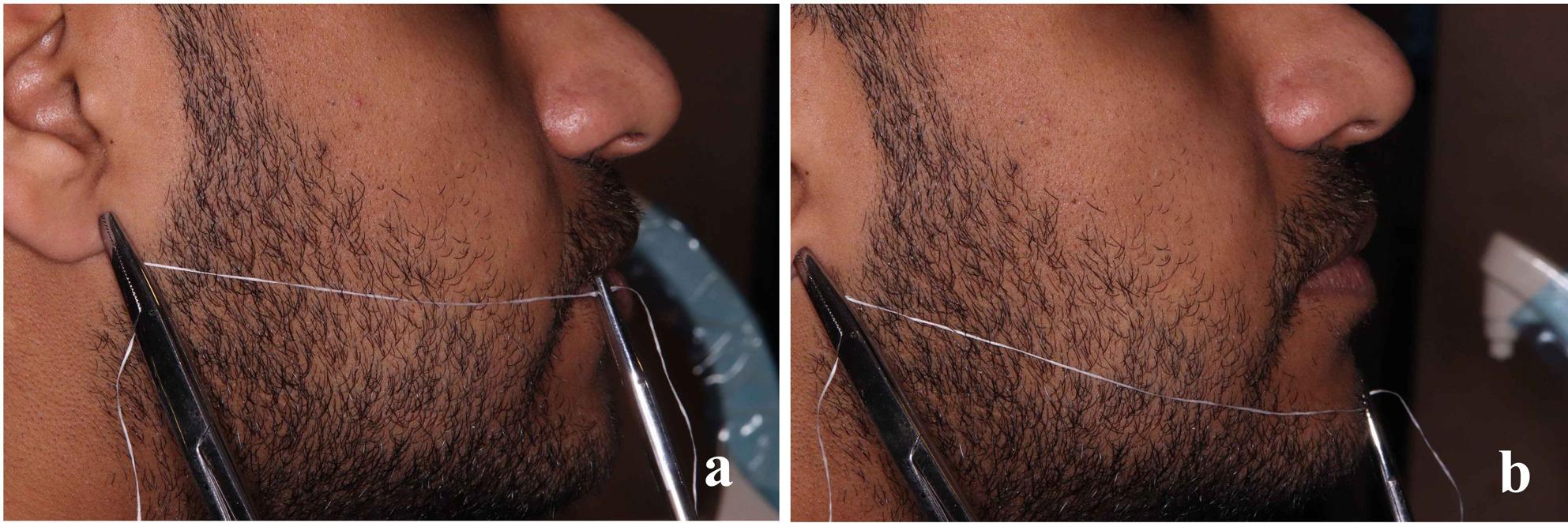
Seventh-day edema: **a**, **b** representative clinical photographs of edema assessment of the same patient in the laser group on the seventh postoperative day

Facial swelling was quantified preoperatively, on the second day, and on the seventh day. Three anatomical landmarks (ear lobule, mandibular angle, and mouth corner) were connected, forming a triangular surface. Distances were measured with a silk thread, and the surface area was calculated. Percentage increase from baseline indicated edema severity. (Figs. 9a, b and 10a, b)

#### Trismus assessment

**Fig. 11:**
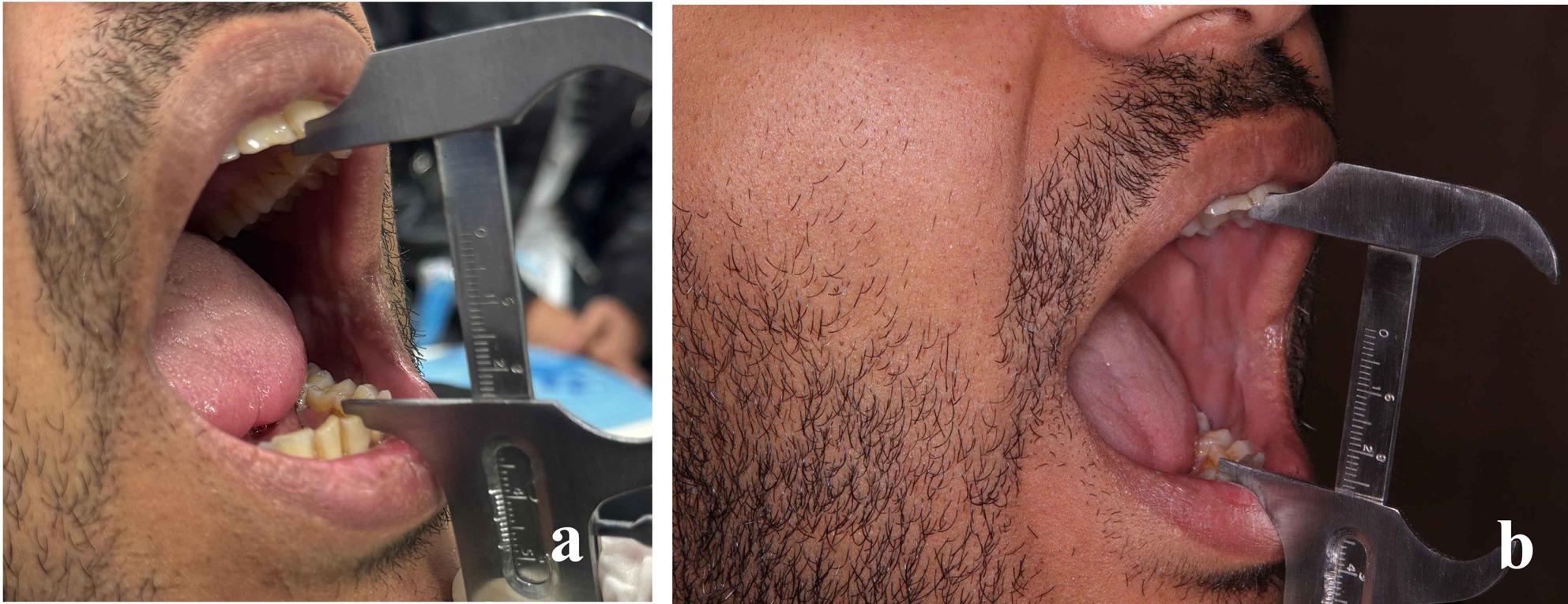
**a** Second-day interincisal opening; **b** Seventh-day interincisal opening

Mouth opening was measured preoperatively, and on days 2 and 7, using a caliper placed between the incisal edges of the upper and lower central incisors at the midline. Reduction in interincisal distance reflected the degree of trismus. (Fig. 11a, b)

#### Wound healing

Clinical assessment was performed on days 2 and 7 for presence or absence of wound dehiscence, persistent bleeding, inflammation, disintegrated clot, or fetid odor.

#### Postoperative complications

Incidence of infection, dry socket, nerve injury, or other adverse events was recorded for both groups.

### Adverse events (harms)

No serious adverse events were observed in either group throughout the study period. No cases of postoperative infection, dry socket, excessive bleeding, allergic reaction, delayed wound healing, or permanent nerve injury were reported. Postoperative pain, edema, and temporary trismus were observed in both groups during the early postoperative period and were managed successfully with the prescribed standard postoperative care without the need for additional interventions or hospital admission. Pain scores were significantly lower in the laser group on postoperative day 2, whereas edema and trismus improved progressively over time in both groups without statistically significant intergroup differences.

#### Radiographic evaluation

**Fig. 12:**
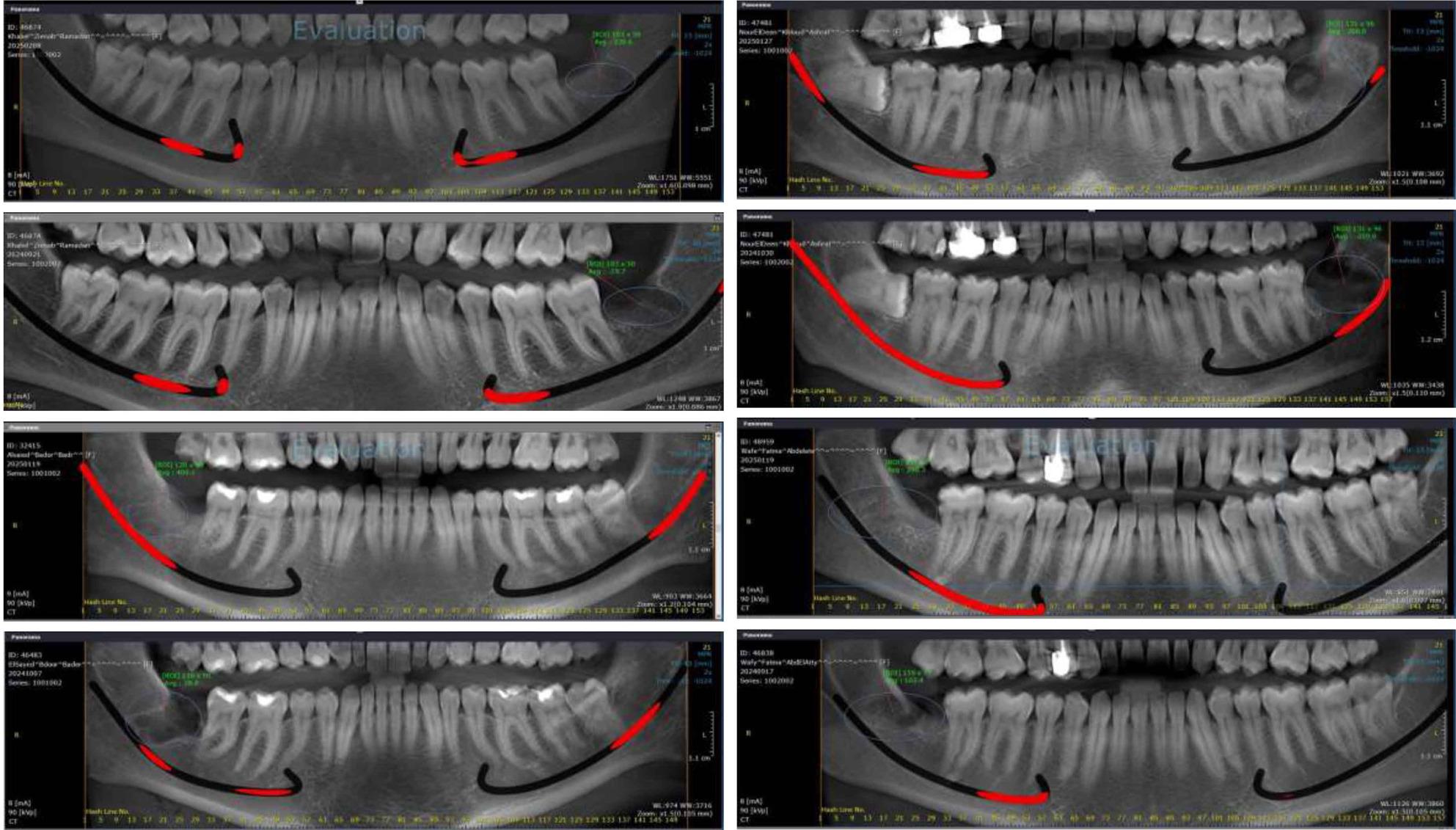
Measuring the bone density

**Fig. 13:**
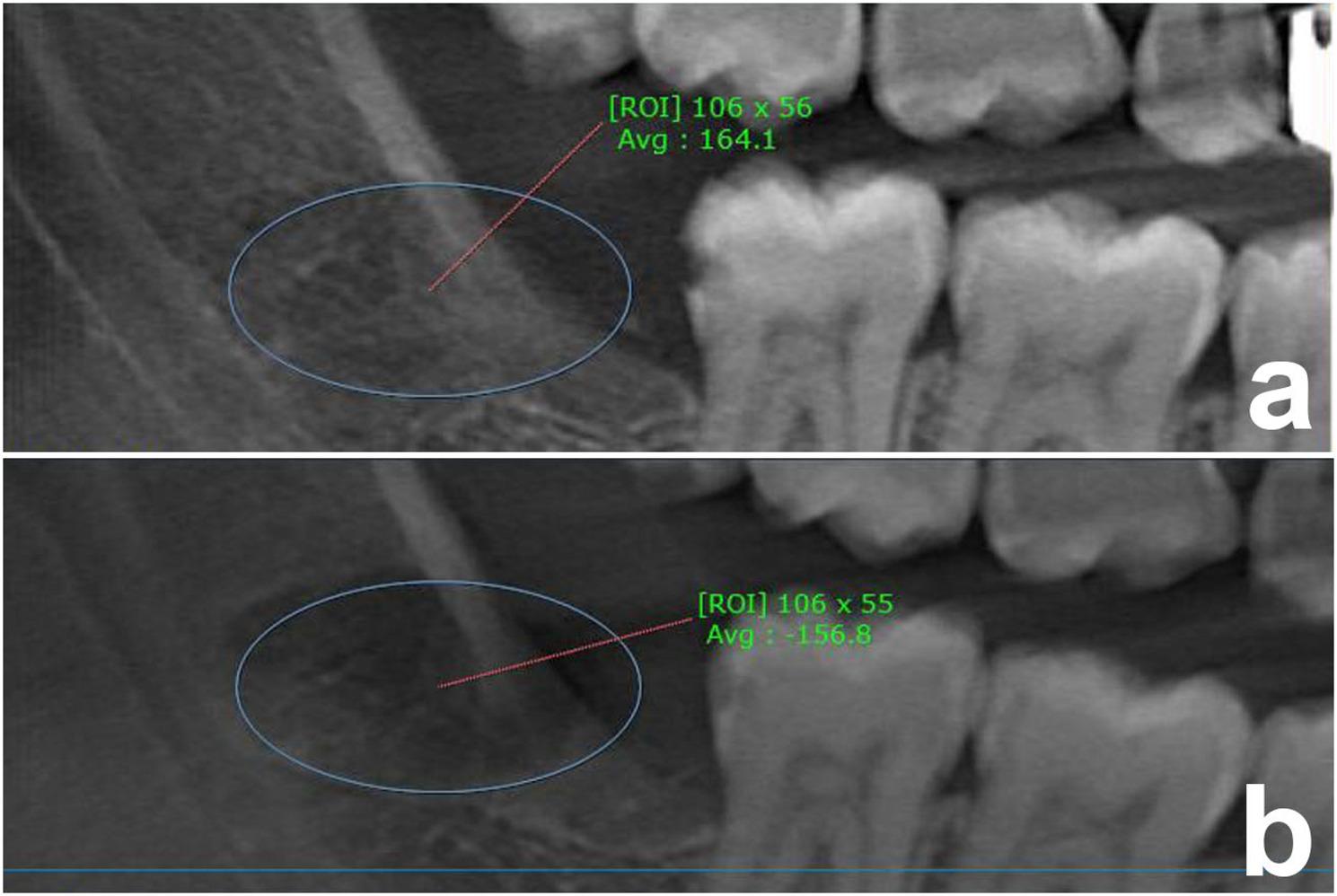
CBCT images demonstrating bone density assessment at the extraction site using a defined region of interest (ROI). **a** Representative CBCT slice showing the selected ROI within the extraction socket with a mean gray value of 164.1; **b** CBCT slice demonstrating ROI-based bone density measurement with a mean gray value of 156.8

**Fig. 14:**
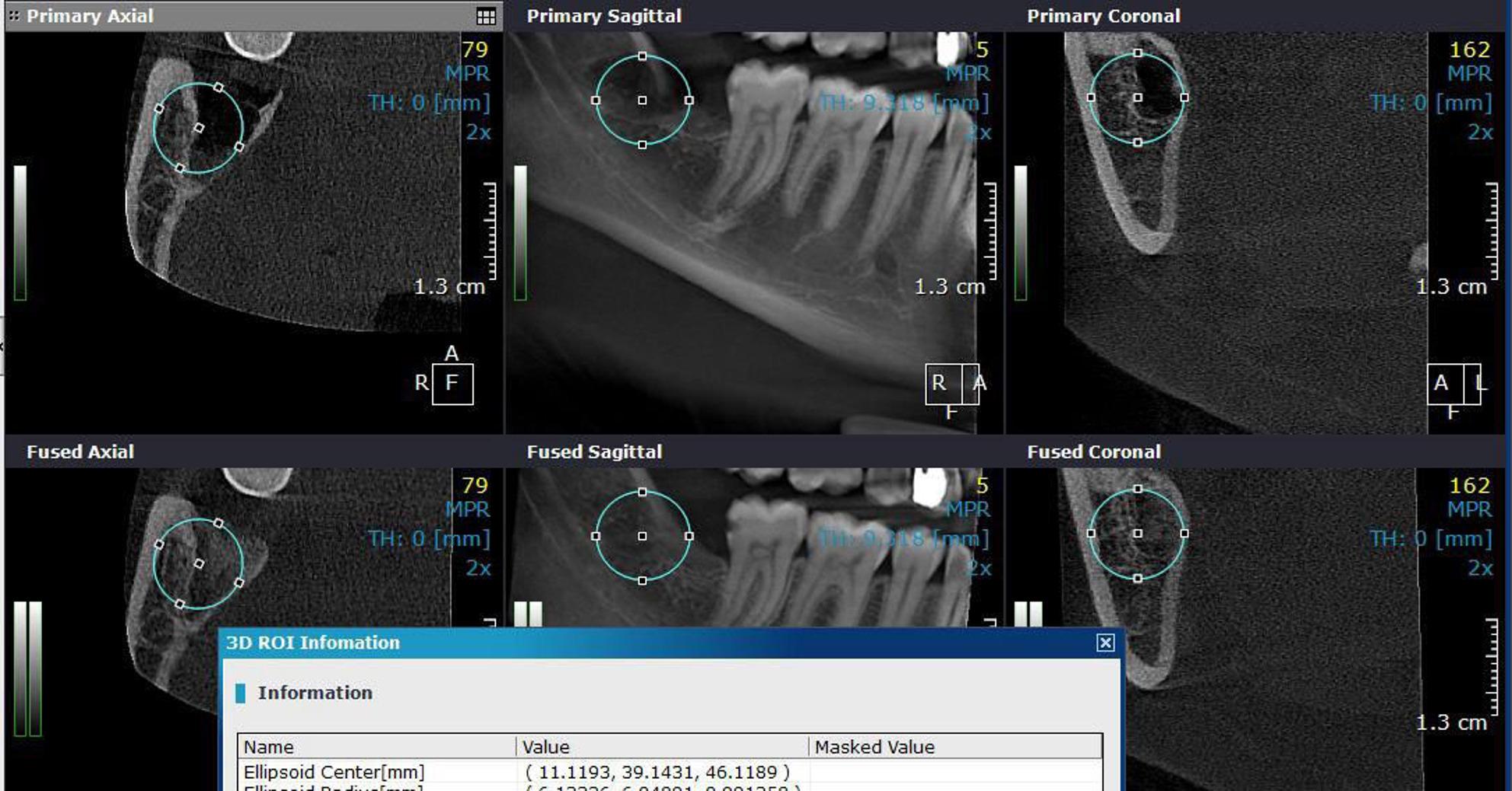
CBCT multiplanar reconstruction images (axial, sagittal, and coronal views) demonstrating the three-dimensional region of interest (ROI) selected within the extraction socket for bone density assessment. The mean gray value and related parameters were calculated using the imaging software

**Fig. 15:**
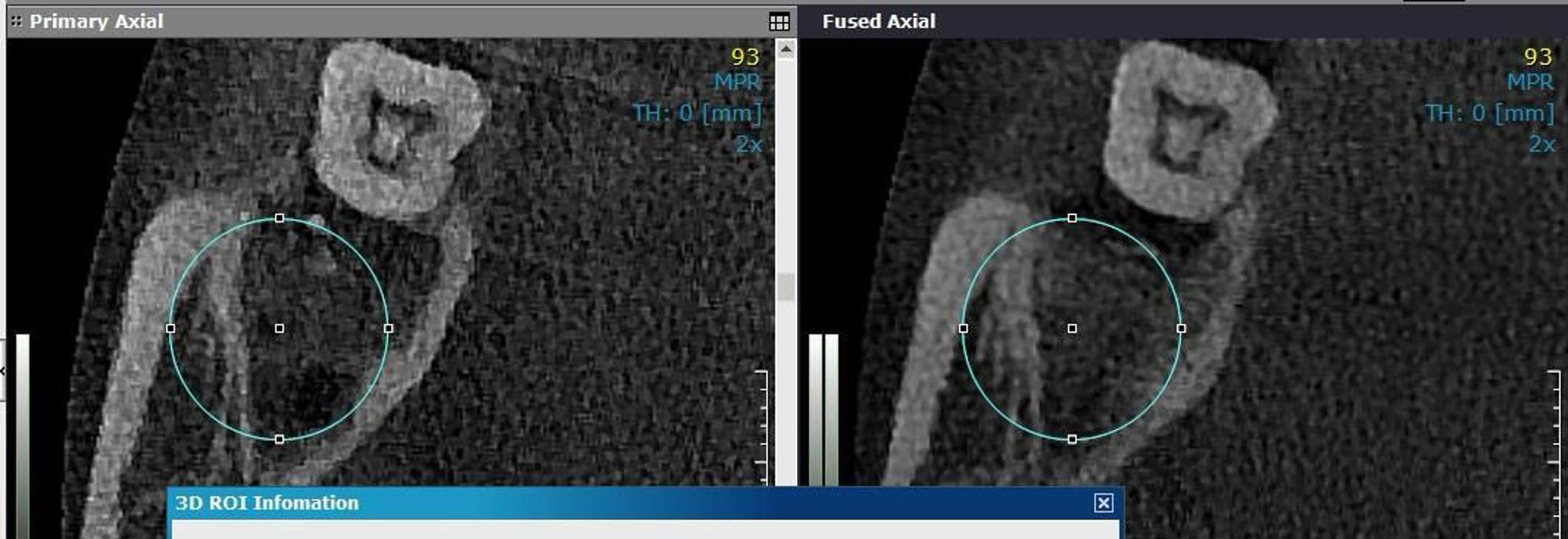
CBCT axial images demonstrating bone density analysis at the extraction site. A three-dimensional region of interest (ROI) was defined within the socket, and the mean gray value was calculated using the imaging software

CBCT imaging was obtained immediately postoperatively and at 3 months to assess bone density changes at the surgical site. Guided by stable anatomical landmarks and the inferior alveolar canal to ensure reproducibility. Identical ROI dimensions and consistent exposure parameters were maintained for all images. Mean grayscale values within each ROI were recorded as relative indicators of bone density, allowing quantitative comparison between preoperative and follow-up stages to monitor radiographic bone healing and remodeling. Bone density at the extraction site was assessed using CBCT imaging. A standardized three-dimensional region of interest (ROI) was defined within the socket, and the mean gray value was calculated using the software analysis tool. (Figs. 12, 13, 14 and 15)

### Operation time

Operation time was recorded for each surgical procedure in two predefined phases: from scalpel incision to final suture placement, and from the start of instrument use to tooth removal. All procedures were performed by the same operator under standardized conditions to ensure consistency.

### Outcome measures

The outcome measures included radiographic bone density, postoperative pain, edema, trismus, and operation time.

### Primary outcome

The primary outcome was radiographic bone density at 3 months postoperatively, assessed using CBCT grayscale values within a standardized ROI. (Figs. 13, 14 and 15)

### Secondary outcomes

- Postoperative pain assessed using a 10-point Visual Analogue Scale (VAS) on postoperative days 2 and 7.
- Facial swelling (edema) measured using standardized linear facial measurements.
- Trismus assessed by maximum interincisal opening (mm).
- Operation time (minutes).

### Statistical analysis

Data were fed into the computer and analyzed using IBM SPSS software package version 27.0. (Armonk, NY: IBM Corp, released in 2020). For continuous data, they were tested for normality by the Shapiro-Wilk test. For normally distributed quantitative variables, data were expressed as mean ± standard deviation (SD). Student’s t-test was used to compare two groups, and ANOVA with repeated measures with Sphericity Assumed was used for comparing the three periods and followed by Post Hoc test (adjusted Bonferroni) for pairwise comparison between each two periods while Paired t-test was used to compare two periods within each group. For non-normally distributed quantitative variables, Mann Whitney test was used to compare two groups while Wilcoxon signed ranks test was used to compare between two periods. In addition, effect size was calculated to estimate the magnitude of differences between the control and study groups. Cohen’s d was used for normally distributed continuous variables while for non-parametric tests, the effect size (r) was calculated. Confidence intervals (C.I) were computed for all effect size measures. Significance of the obtained results was judged at the 5% level, all tests were two-tailed. (Table 2)

**Table 2:** Normality check using Shapiro-Wilk test for different parameters

Measures	Groups	Time	Shapiro-Wilk		
			Statistic	df	Sig.
Trismus	**Control**	**Pre**	0.901	12	0.165
		**2nd day**	0.981	12	0.989
		**7th day**	0.974	12	0.945
	**Study**	**Pre**	0.872	12	0.069
		**2nd day**	0.942	12	0.521
		**7th day**	0.893	12	0.130
Swelling	**Control**	**Pre**	0.977	12	0.970
		**2nd day**	0.927	12	0.347
		**7th day**	0.929	12	0.373
	**Study**	**Pre**	0.940	12	0.495
		**2nd day**	0.938	12	0.474
		**7th day**	0.965	12	0.855
Pain	**Control**	**2nd day**	0.866	12	0.059
		**7th day**	0.802	12	0.010^*^
	**Study**	**2nd day**	0.870	12	0.066
		**7th day**	0.802	12	0.010^*^
Bone density	**Control**	**Immediate post-operative**	0.852	12	0.039^*^
		**3 months**	0.919	12	0.279
	**Study**	**Immediate post-operative**	0.765	12	0.004^*^
		**3 months**	0.952	12	0.670
Increase in Bone density	**Control**	**–**	0.837	12	0.025^*^
	**Study**	**–**	0.966	12	0.868
Operation time (min) scalpel to final suture	**Control**	**–**	0.969	12	0.899
	**Study**	**–**	0.970	12	0.911
Operation time (min) instrument use to removal	**Control**	**–**	0.975	12	0.958
	**Study**	**–**	0.928	12	0.356

*: Statistically significant at *p* ≤ 0.05

## Results

A total of 24 patients were enrolled in this randomized controlled clinical trial and were equally allocated into two groups: control (*n* = 12) and study (*n* = 12). All patients completed the follow-up period and were included in the final analysis.

The baseline characteristics of the studied groups are presented in Table 3. No statistically significant differences were found between the two groups regarding preoperative variables (*p* > 0.05), indicating baseline comparability between the groups.  

**Table 3:** Baseline characteristics of the studied groups

Variable	Control (*n* = 12)	Study (*n* = 12)	Test	*p*-value
Age (years)	24.8 ± 3.5	25.3 ± 3.7	t = 0.36	0.721
Sex (Male/Female)	7/5	6/6	χ^2^ = 0.17	0.680
Infection	6/6	5/7	χ^2^ = 0.17	0.680
Preoperative mouth opening (mm)	42.0 ± 3.13	42.25 ± 6.93	t = 0.11	0.911
Preoperative swelling	14.10 ± 1.19	14.13 ± 1.22	t = 0.05	0.960

Data are expressed as mean ± standard deviation (SD) or number (frequency)

t: Student’s t-test; χ²: Chi-square test

*p* ≤ 0.05 is considered statistically significant

### Bone density

Bone density was assessed radiographically using cone-beam computed tomography (CBCT). Scans were obtained immediately after surgery and at the 3-months follow-up using the same CBCT unit and exposure parameters for all patients. Bone density was quantified by measuring grayscale voxel values within a standardized three-dimensional ellipsoid volume of interest (VOI) positioned at the center of the osteotomy site. The size and location of the VOI were kept constant across all scans to ensure reproducibility. Measurements were performed twice by the same calibrated examiner, and the mean of the two readings was recorded. Higher grayscale values indicated higher bone density. (Table 4, Figs. 12, 13, 14 and 15)

**Table 4:** Comparison between the two studied groups according to bone density

Bone density	Control (*n* = 12)	Study (*n* = 12)	U	*p*	Effect Size *r* (95% C.I)
Immediate	160.0 (150.3–170.00)	154.0 (146.0–162.5)	54.00	0.319	0.213 (-0.209–0.567)
3 months	270.0 (213.2–293.3)	310.7 (282.3–346.0)	31.50*	0.017^*^	0.477 (0.092–0.739)
Change (Increase)	114.3 (49.40–134.8)	163.7 (113.0–189.3)	31.00^*^	0.017^*^	0.483 (0.099–0.742)
Z, p_0_	Z = 3.059^*^, p_0_ = 0.002^*^	Z = 3.059^*^, p_0_ = 0.002^*^			

Data was expressed using Median with Inter quartile range (IQR) U: Mann Whitney test

*CI:* Confidence interval Z: Wilcoxon signed ranks test

p: *p* value for comparing between the two studied groups

p_0_: *p* value for comparing between Immediate and 3 months in each group

*: Statistically significant at *p* ≤ 0.05

### Pain

Pain intensity was evaluated using a 10-cm Visual Analogue Scale (VAS), where “0” indicated no pain and “10” represented the worst pain imaginable. Patients were instructed to mark their pain level on the scale at each assessment point. Pain measurements were recorded on postoperative day 2 and day 7. All patients received Paracetamol 500 mg three times daily as the standard analgesic regimen unless contraindicated, and no additional analgesics were prescribed to avoid interference with postoperative pain assessment. (Table 5)

**Table 5:** Comparison between the two studied groups according to Pain

Pain	Control (*n* = 12)	Study (*n* = 12)	U	*p*	Effect Size *r* (95% C.I)
2nd day	3.0 (2.50–4.50)	2.0 (1.0–3.0)	32.50^*^	0.020^*^	0.480 (0.095–0.740)
7th day	1.0 (0.0–1.50)	0.50 (0.0–1.50)	69.00	0.887	0.038 (-0.371–0.435)
Z, p_0_	Z = 3.081^*^, p_0_ = 0.002^*^	Z = 2.584^*^, p_0_ = 0.010^*^			

Data was expressed using Median with Inter quartile range (IQR) U: Mann Whitney test

*CI:* Confidence interval Z: Wilcoxon signed ranks test

p: *p* value for comparing between the two studied groups

p_0_: *p* value for comparing between 2nd day and 7th day in each group

*: Statistically significant at *p* ≤ 0.05

### Swelling

Swelling was assessed using a standardized facial linear-measurement protocol. Three distances were measured on the operated side using a flexible millimeter tape: (1) from the lateral canthus of the eye to the angle of the mandible, (2) from the tragus to the corner of the mouth, and (3) from the tragus to the soft-tissue pogonion. The sum of these three measurements represented the overall swelling value for each patient. All measurements were recorded preoperatively, on postoperative day 2, and on postoperative day 7 by the same examiner to ensure consistency. (Table 6)

**Table 6:** Comparison between the two studied groups according to Swelling

Swelling	Control (*n* = 12)	Study (*n* = 12)	t	*p*	Effect Size Cohen’s d (95% C.I)
Pre	14.10^b^ ± 1.19	14.13^b^ ± 1.22	0.051	0.960	0.021 (-0.780–0.821)
2nd day	16.26^a^ ± 1.21	16.33^a^ ± 1.03	0.163	0.872	0.067 (-0.734–0.866)
7th day	15.53^ab^ ± 1.25	15.0^b^ ± 1.14	1.073	0.295	0.438 (-0.377–1.244)
F, p_0_	F = 11.111^*^ p_0_ < 0.001^*^	F = 11.279^*^ p_0_ < 0.001^*^			

Data was expressed using Mean ± Standard deviation (SD); t: Student's t-test

*CI:* Confidence interval

p: *p* value for comparing between the two studied groups

F: F test (ANOVA) with repeated measures with Sphericity Assumed, Sig. bet. periods was done using Post Hoc Test (adjusted Bonferroni)

p_0_: *p* value for comparing between the three studied periods in each group

*: Statistically significant at *p* ≤ 0.05

Different superscript letters (a, b) indicate statistically significant differences between periods within the same group (Bonferroni post hoc test), whereas identical or shared superscript letters (e.g., ab) indicate no statistically significant difference

### Trismus

Trismus was evaluated by recording the maximum inter-incisal distance using a calibrated digital Vernier caliper. Patients were instructed to open their mouths as widely as possible without discomfort, and the distance between the incisal edges of the upper and lower central incisors was measured in millimeters. Trismus measurements were obtained at the same time points as the swelling assessment (Preoperative, day 2, and day 7). (Table 7)

**Table 7:** Comparison between the two studied groups according to trismus

Trismus	Control (*n* = 12)	Study (*n* = 12)	t	*p*	Effect Size Cohen’s d (95% C.I)
Pre	42.0^a^ ± 3.13	42.25^a^ ± 6.93	0.114	0.911	0.047 (-0.754–0.846)
2nd day	27.04^c^ ± 4.28	31.17^b^ ± 5.77	1.990	0.059	0.812 (-0.031–1.639)
7th day	34.63^b^ ± 3.66	35.50^ab^ ± 6.43	0.410	0.687	0.167 (-0.636–0.967)
F, p_0_	F = 50.594^*^, p_0_ < 0.001^*^	F = 6.822* p_0_ = 0.005^*^			

Data was expressed using Mean ± Standard deviation (SD); t: Student's t-test

*CI:* Confidence interval

p: *p* value for comparing between the two studied groups

F: F test (ANOVA) with repeated measures with Sphericity Assumed, Sig. bet. periods was done using Post Hoc Test (adjusted Bonferroni)

p_0_: *p* value for comparing between the three studied periods in each group

*: Statistically significant at *p* ≤ 0.05

Different superscript letters (a, b, c) indicate statistically significant differences between periods within the same group (Bonferroni post hoc test), whereas identical or shared superscript letters (e.g., ab) indicate no statistically significant difference

### Operation time

Operation time was significantly longer in the study (laser) group compared to the control (rotary) group (*p* < 0.001), with a very large effect size, indicating a marked difference between the two techniques. (Table 8)

**Table 8:** Comparison between the two studied groups according to operation time (min)

Operation time (min)	Control (*n* = 12)	Study (*n* = 12)	t	*p*	Effect Size Cohen’s d (95% C.I)
From Scalpel to final suture	13.0 ± 3.57	35.25 ± 5.10	12.382^*^	< 0.001^*^	5.055 (3.356–6.726)
From start of instrument use to removal	8.0 ± 1.71	22.75 ± 4.27	11.120^*^	< 0.001^*^	4.540 (2.971–6.079)

Data was expressed using Mean ± Standard deviation (SD); t: Student's t-test

*CI:* Confidence interval

p: *p* value for comparing between the two studied groups

*: Statistically significant at *p* ≤ 0.05

## Discussion

The present randomized clinical trial evaluated the clinical and radiographic outcomes of Er:YAG laser osteotomy compared with conventional rotary instrumentation for the removal of impacted mandibular third molars. The findings suggest that the laser technique was associated with lower postoperative pain scores and higher radiographic grayscale values at the 3-months follow-up. Edema and trismus improved over time in both groups, although no statistically significant intergroup differences were observed [24].

Regarding clinical outcomes, patients treated with the Er:YAG laser experienced lower postoperative pain and swelling, particularly during the early postoperative period [25]. These findings may be attributed to the laser’s precise micro-ablative action, minimal thermal damage, and reduced mechanical trauma to surrounding tissues. Similar observations have been reported in previous studies, which demonstrated improved patient comfort and reduced inflammatory response following laser-assisted osteotomy [26, 27].

In terms of radiographic outcomes, the laser group demonstrated higher bone density at the 3-months follow-up. This may reflect more favorable radiographic bone density changes associated with reduced necrotic bone formation and improved osteoblastic activity following laser application. These findings are consistent with previous reports suggesting potential regenerative benefits of laser osteotomy [28, 29]. However, it should be noted that CBCT grayscale values represent relative measurements rather than absolute Hounsfield units. In the present study, all scans were standardized using identical acquisition parameters, allowing reliable intra-study comparisons.

A notable finding was the longer operation time observed in the laser group. This may be explained by the lower ablation efficiency of the laser compared with rotary instruments, in addition to the technique sensitivity associated with laser application. Although prolonged surgical time may theoretically increase tissue exposure and postoperative risk, the improved clinical outcomes observed in this study suggest that this did not adversely affect healing. Nevertheless, this factor should be considered when selecting the appropriate surgical approach [30, 31].

Overall, the findings of this randomized clinical trial suggest that Er:YAG laser osteotomy is associated with improved selected clinical outcomes, particularly postoperative pain, in addition to higher radiographic grayscale values compared with conventional rotary instrumentation. These results support the potential role of laser-assisted osteotomy as a valuable alternative in third molar surgery [32]. However, these findings should be interpreted with caution in light of the study limitations, and further well-designed studies with larger sample sizes and longer follow-up periods are warranted to confirm these outcomes [33].

### Limitations

This study has several limitations. First, the relatively small sample size may limit the generalizability of the findings. Second, the retrospective trial registration may raise concerns regarding transparency, although all outcomes were predefined in the study protocol. Third, the use of standardized postoperative analgesics may have partially masked differences in pain perception between groups. Additionally, the surgical procedure is inherently operator-dependent, which may introduce variability despite protocol standardization. Finally, the follow-up period was limited to three months and may not fully reflect long-term bone remodeling.

Overall, within the limitations of this study, Er:YAG laser osteotomy suggested clinical advantages in reducing postoperative morbidity and improving early radiographic bone healing compared with conventional rotary techniques.

## Conclusions

Within the limitations of this randomized controlled clinical trial, Er:YAG laser osteotomy was associated with lower postoperative pain and higher radiographic bone density (grayscale values) at 3 months compared with conventional rotary osteotomy during impacted mandibular third molar removal. Edema and trismus improved over time in both groups, with no statistically significant intergroup differences observed. Both techniques were clinically effective for third molar surgery. Further studies with larger sample sizes and longer follow-up periods are required to validate these findings.

## Supplementary Information

Supplementary Material 1.

Supplementary Material 2.

Supplementary Material 3.

Supplementary Material 4.

## Data Availability

The datasets utilized and/or analyzed during this study is available from the corresponding author upon reasonable request.

## Abbreviations

ANOVA: Analysis of variance
ASA: American Society of Anesthesiologists
CBCT: Cone-beam computed tomography
Er:YAG: Erbium-doped Yttrium Aluminium Garnet
ROI: Region of interest
VAS: Visual analogue scale
