# Supplementary material for: Efficacy of Er:YAG laser in removal of impacted mandibular third molars (a randomized controlled clinical trial)
Source: BMC Oral Health. 2026 Jun 5;26:1060. doi: 10.1186/s12903-026-08790-w (PMC13270853; doi:10.1186/s12903-026-08790-w)
Supplement: Supplementary file 1 — Supplementary Material 1. [file 12903_2026_8790_MOESM1_ESM.pdf]

لجنة
أخلاقيات البحث العلمى

جامعة الإسكندرية
ALEXANDRIA
UNIVERSITY

كلية طب الأسنان
قسم: ماجستير الإكلينيكى لتطبيقات الليزر فى طب الأسنان

خطة المقترح البحثي لنيل درجة ماجستير الإكلينيكى لتطبيقات الليزر فى طب الأسنان

تحت عنوان

Efficacy of ER: YAG LASER in removal of impacted mandibular third molars
(A Randomized Controlled Clinical trial)

كفاءة الليزر المائى في إزالة ضروس العقل المدفونة بالفك السفلي
(تجربة إكلينيكية عشوائية محكمة)

العام الجامعي ٢٠٢٣/٢٠٢٤

اسم الباحث : كريم أنور أحمد نظمى

المشرف الرئيسي : أ.د/ أحمد عادل عبد الحكيم

جامعة الإسكندرية
ALEXANDRIA
UNIVERSITY

Faculty of Dentistry
Department: Clinical Master Degree in Oral Laser Applications

Student Code No.

Protocol for M. Sc. Degree

In Clinical Master Degree in Oral Laser Applications
Academic Year 2023-2024

Name of Candidate: Karim Anwer Ahmed Nazmy

Title:

English Title: **Efficacy of ER:YAG LASER in removal of impacted mandibular third molars (A Randomized Controlled Clinical trial)**

Arabic Title: **كفاءة الليزر المائى في إزالة ضروس العقل المدفونة بالفك السفلي (تجربة إكلينيكية عشوائية محكمة)**

Keywords: ER:YAG LASER, impacted mandibular third molars, surgical removal (rotary)

Supervision Committee:

1- **Prof. Dr. Ahmed Adel Abdelhakim**
Professor of Prosthodontics
Faculty of Dentistry
Alexandria University

2- **Prof. Josep Arnabat Dominguez**
Associate Professor of Oral Surgery
Faculty of Dentistry
Barcelona University

JOSEP ARNABAT DOMINGUEZ - DNI 77280789F
Firmado digitalmen[illegible] por JOSEP ARNABA[illegible] DOMINGUEZ - DNI 77280789F
Fecha: 2024.09.11 19:13:41 +02'00'

3- **Prof. Dr. Riham Eldibany**
Professor of Oral and Maxillofacial Surgery
Faculty of Dentistry
Alexandria University

Role of Supervisors

1. **Dr. Ahmed Adel Abdelhakim**

   Supervision of the clinical procedures

   Conceptualization/formulation of study plan and design of the study.

   Analysis and interpretation of study results.

   Supervision of study execution and writing.

   Revising the thesis.

2- Prof. Josep Arnabat Dominguez

Supervision of the clinical procedures

Conceptualization/formulation of study plan and design of the study.

Analysis and interpretation of study results.

Supervision of study execution and writing.

Revising the thesis.

3. **Dr. Riham Moustafa Eldibany**

   Supervision of the clinical procedures

   Conceptualization/formulation of study plan and design of the study.

   Analysis and interpretation of study results.

   Supervision of study execution and writing.

   Revising the thesis.

CONTENTS

Contents	Page
ABSTRACT	1
INTRODUCTION	2
AIM OF THE STUDY	4
MATERIALS AND METHODS	5
STATISTICAL ANALYSIS	14
ETHICAL CONSIDERATIONS	15
ESTIMATED BUDGET	16
DURATION OF THE STUDY	17
ANTICIPATED PROBLEMS	18
PUBLICATION POLICY	19
REFERENCES	20

ABSTRACT

Background: Efficacy of ER:YAG LASER in removal of impacted mandibular third molars.

Aim: This study aims to assess the postoperative complications following the removal of impacted mandibular third molars using Er:YAG LASER and conventional handpiece drilling system.

Materials: Er:YAG LASER, conventional handpiece, surgical burs and elevators.

Methods: preoperative phase, operative phase, post operative phase and follow up phase.

Analysis: The data will be processed and analyzed using statistical package for social sciences program SPSS software. The study will include descriptive and analytical data. An a-P value of less than 0.05 will be considered statistically significant.

Keywords: Er:YAG LASER, impacted mandibular third molars, surgical removal (rotary)

INTRODUCTION

Surgical removal of impacted mandibular third molars is one of the most common outpatient surgical procedures. It is also the most commonly performed procedure in oral and maxillofacial surgery around the world. Nearly all patients undergoing surgical removal develop some degree of pain, swelling, and trismus after the surgery, and there seems to be no consensus on the best perioperative treatment to minimize these complications.(1)

Osteotomy and ostectomy are often needed to remove retentive bone around third molars in order to facilitate extractions.(2) It is essential to perform an atraumatic osteotomy to access teeth and promote healing.(3)

Rotary systems have been used for many years to accomplish this, but there are several problems that persist despite technological developments. These problems include bone debris accumulation, overheating of surrounding bone, with subsequent necrosis, vibration, and patient discomfort.(4)

LASER stands for “light amplification by the stimulated emission of radiation.” The instrument creates light energy in a very narrow and focused beam. This laser light produces a reaction when it hits tissue, allowing it to remove or shape the tissue.(5)

Laser dentistry is the use of lasers to treat a number of different dental conditions. It became commercially used in clinical dental practice for procedures involving tooth tissue in 1989.(5)

Laser dentistry potentially offers a more comfortable treatment option for a number of dental procedures involving hard or soft tissue compared to drills and other non-laser tools.(5)

Erbium yttrium– aluminum–garnet (Er: YAG) LASER has a wavelength of 2940 nm, which is absorbed by hydroxyapatite and water. Therefore, the main components of bone, including organic matrix and inorganic calcium salts, are the targets for the Er: YAG laser beam. Such laser reduces significantly in bone temperature than conventional rotary tools and penetrates only 0.1mm in the hard tissue, providing a safe, precise, and minimally invasive action.[(6)]

Further advantages of Er:YAG action on soft and hard tissues are their bactericidal and the bio stimulating effects, which seem to accelerate the healing process. A clean surgical field, following bone evaporation is another key factor of Er:YAG use.[(7)]

The null hypothesis of this study purposes that there will be no significant difference in postoperative complications following the removal of impacted mandibular third molars using Er:YAG LASER or conventional handpiece drilling system.

AIM OF THE STUDY

General aim

This study aims to assess the efficacy of using Er:YAG LASER in removal of impacted mandibular third molars and conventional handpiece drilling system.

Specific aim

- Compare post-operative pain, edema and trismus.
- Compare bone density radiographically by CBCT.

MATERIALS AND METHODS

Study design

This study will be a randomized parallel comparative clinical study.

Twenty four patients of both genders having mesioangular bony impacted mandibular third molars will be selected.

In the study group bone removal will be performed using Er:YAG LASER while for the control group bone removal will be performed using conventional handpiece drilling system.

Patients having mesioangular bony impacted mandibular third molars

In the study group bone removal will be performed using Er:YAG

Conventional handpiece drilling system

Assessment of post operative complications

Study setting

This prospective study will be conducted on 24 patients with impacted mesioangular mandibular third molars, who visit the outpatient clinic of Oral and Maxillofacial Surgery Department, Faculty of dentistry, Alexandria University for removal of impacted mandibular third molar.

Participants

Study sample and Sample size estimation

Sample size was estimated based on assuming 95% confidence level and 80% study power.(8) Based on difference between two independent means, a sample of 12 patients per group is required yielding effect size of 1.211. Total sample size = number per group x number of groups = 12 x24 patients.

Sample size was based on Rosner's method(9) calculated by G* Power 3.0.10.(10)

Randomization and Allocation Concealment

Participants will be randomly allocated into a study group and a control group using computer generated random list. The participant allocation lists will be kept in opaque, sealed envelopes and arranged sequentially by a dental assistant who will not be involved in the study. Each envelope will be opened at the time of intervention.

Patients will be divided intro 2 equal groups:

Study group: 12 patients having impacted mesioangular mandibular third molar teeth, where bone removal will be performed using Er:YAG LASER.

Control group: 12 patients having impacted mesioangular mandibular third molar teeth, where bone removal will be performed using conventional handpiece drilling system.

Patients' inclusion criteria:

1. Patients with an indication for surgical extraction of the included lower mesioangular impacted third molar who undergoes an ostectomy and/or dental section.
2. Age between 18-45 years of both genders.
3. Absence of associated pathology in the teeth adjacent to the included third molars.
4. Patients who cooperates with the study, complies with the postoperative follow-up and signs the informed consent.
5. ASA class I and II.

Exclusion criteria

- Patients with systemic diseases (ASA III or higher) that contraindicate surgical intervention or that impair wound healing.

- Patients with antibiotic pre-medication or under any pharmacological treatment that interferes with wound healing.
- Patients who are under chronic treatment with NSAIDs.
- Patients with acute pericoronitis or severe periodontal disease.
- Patients in whom the administration of medication or local anesthesia of the study protocol is contraindicated.
- Patients who undergo the extraction of another tooth in the same surgical location.

Materials

Device:

Er:YAG: LASER (Erbium-doped Yttrium Aluminium Garnet LASER, Erbium YAG LASER*) is a solid-state LASER whose active LASER medium is erbium-doped yttrium aluminium garnet (Er:Y3Al5O12). Er:YAG LASERS typically emit light with a wavelength of 2940 nm, which is infrared light.

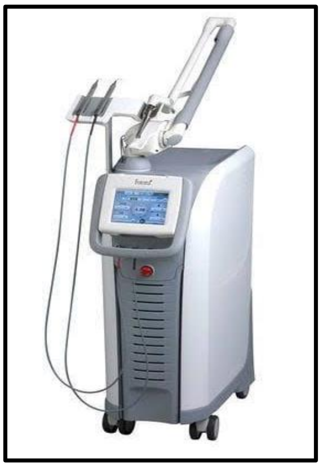

* LightWalker AT/AT S®, Fontona. Europe.

METHODS

Pre-operative phase

A- Clinical Examination

- Complete history taking including: name, age, occupation, residence, chief complain and dental history from all patients.
- Patients intraoral examination to determine the condition of the soft tissue covering the impacted third molar, and the condition of the pericoronal flap: healthy, inflamed or infected.
- Measuring interincisal opening and outer contour of the cheek before and after surgery.

B- Radiographic examination

- Panoramic radiography machine type for all patients to evaluate the classification of the impacted third molars.
- Cone beam computed tomography (CBCT) to evaluate the amount of bone covering the impacted tooth, root patterns, proximity to the adjacent tooth and relation to inferior alveolar nerve.

Operative phase

Surgical Procedure

- All operations will be performed under local anesthesia in the LASER Clinic, Faculty of Dentistry, Alexandria University.
- All patients will be given local anesthetic (Mepevcaine-L) with direct technique for inferior alveolar nerve block and buccal infiltration.
- Making the mucoperiosteal flap using no. 15 blade mounted on Bard Parker scalpel handle starting just lingual to the external ridge of the ramus of the mandible at a distance of 1.5 cm distal from the lower second molar and directing anteriorly until the midpoint of the surface of the second molar.

The incision will continue buccally around the neck of the second molar to the interproximal space between the first and the second molars then extended down obliquely toward the mucobuccal fold at a 45 degrees angle.

- Then gently reflect flap with a periosteal elevator.

Study group

- Bone removal will be accomplished using a Er:YAG LASER Buccal and distal guttering will be performed then making a diagonal split of the interlocked mesial cusp, then a suitable elevator will be applied mesially at the neck of the tooth and rotated to move it distally and occlusally.
- Removing any remnants of the tooth follicle by the mean of a curette.
- Trimming and filing of irregular bony edges with a bone file.
- Irrigating the wound with saline.
- Repositioning the flap, suturing the edges using 000 black silk suture, and applying a pressure pack.

Control group

- Bone removal will be accomplished using a fissure surgical bur, Buccal and distal guttering will be performed then making a diagonal split of the interlocked mesial cusp, then a suitable elevator will be applied mesially at the neck of the tooth and rotated to move it distally and occlusally.
- Removing any remnants of the tooth follicle by the mean of a curette.
- Trimming and filing of irregular bony edges with a bone file.
- Irrigating the wound with saline.
- Repositioning the flap, suturing the edges using 000 black silk suture, and applying a pressure pack.

Post-operative phase

- Both groups will be given an oral antibiotic (Amoxicillin&Clavulanate Potassium* 1gm. twice daily for four days) to decrease the possibility of post-operative infection.
- Both groups will be given analgesics (Paracetamol 500 mg Tablets** twice daily) for three days to relief pain.
- All patients will be instructed to apply ice bag extraorally four times on the first 6 hours after surgery.
- Soft diet for the first 24 hours after surgery.
- Rinsing the mouth with a warm antiseptic mouth wash starting on the second post-operative day and lasting for a week.
- Removing the sutures after 7 days.

Clinical Follow-up

1) Post-operative pain

Pain evaluation at the second and seventh day postoperatively through the visual analogue scale (VAS).(11) Patients are asked about the pain severity according to (VAS) as follow:

0- No pain - The patient feels well.

1- Slight pain - If the patient is distracted he or she does not feel the pain.

2 - Mild pain - The patient feels the pain even if on some activity.

3- Severe pain - The patient is very disturbed but nevertheless can continue with normal activities.

4- Very severe pain -The patient is forced to abandon normal activities.

5- Extremely severe pain - The patient must abandon every type of activity and feels the need to lie down.

---

* Augmentin: British, GlaxoSmithKline

** Panadol: GSK group, UK.

2) Post-operative edema

Edema will be evaluated while the patient is sitting in an upright position. The measurements are taken between three fixed points on the face; the lobule of the ear, the angle of the mandible and the corner of the mouth. These points represent a triangle. The distance between two points is measured using 000 black silk threads that are clamped at each end by a mosquito forceps opposite to the point.

The data will be collected preoperatively and at the second and seventh days postoperatively. The surface area of this triangle is calculated; the percentage of increase in this triangular area indicates the degree of swelling.

3) Post-operative trismus

Trismus will be evaluated pre-operatively and post-operatively at the second and seventh days through measuring the maximum mouth opening using a caliper applied between the incisal edges of the upper central incisors and the lower central incisors at the midline.

4) Post-operative healing

Healing will be evaluated clinically at the second and seventh days postoperatively regarding to the presence or absence of wound dehiscence, bleeding, inflammation, any disintegrated clot and fetid odour.

5- Presence or absence of post-operative complications

Follow up phase

- Radiographic Follow-up

Cone beam computed tomography (CBCT) will be done for each patient immediately and after 3 months postoperative to assess bone density.

Flow Chart

Study design: Randomized parallel comparative clinical study

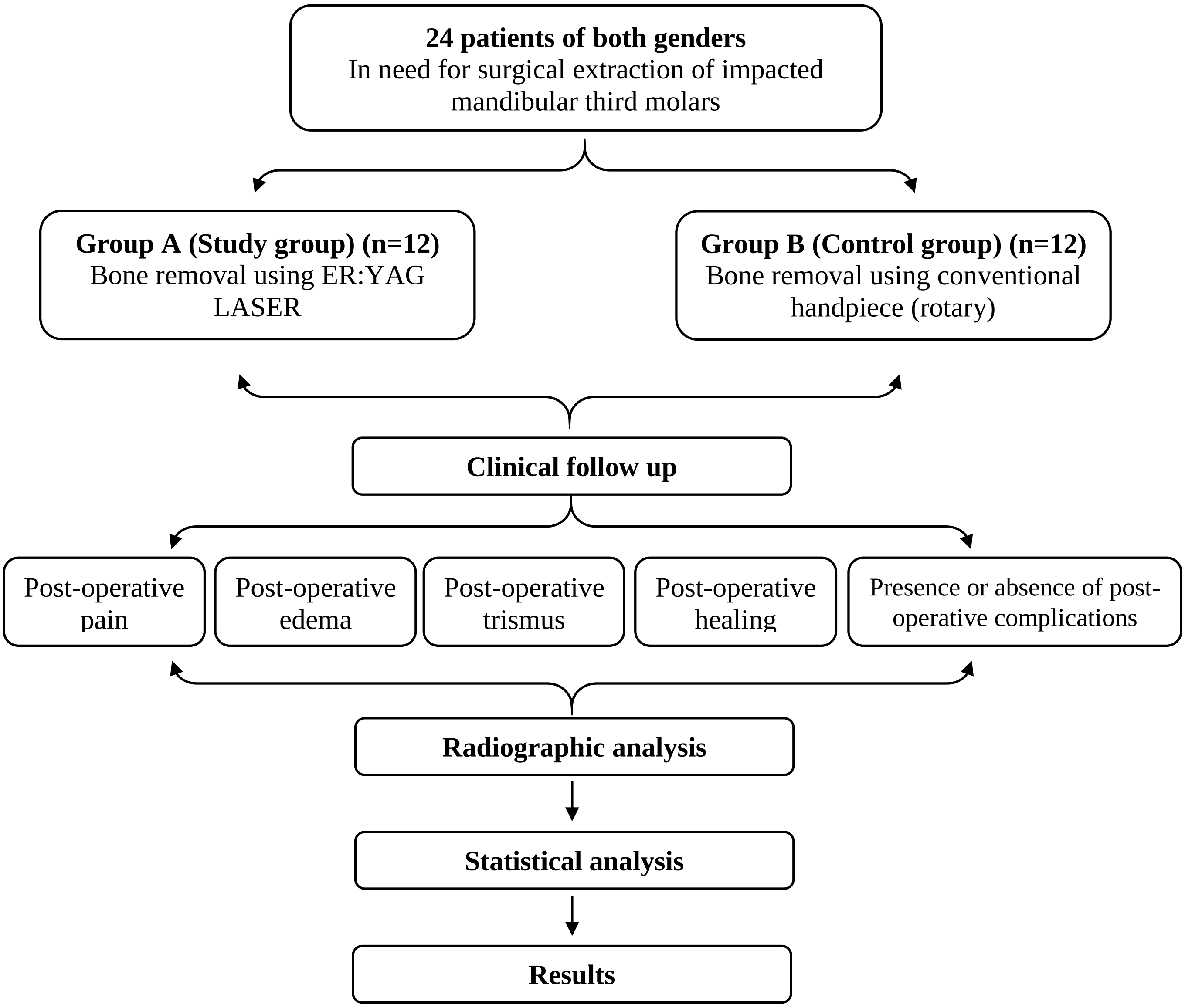

STATISTICAL ANALYSIS

The data will be processed and analyzed using statistical package for social sciences program SPSS software. The study will include descriptive and analytical data. An a-P value of less than 0.05 will be considered statistically significant.

ETHICAL CONSIDERATIONS IN SUBJECTS INVOLVING HUMAN PARTICIPANTS

1. Statement that the research protocol will be approved by the Research Ethics Committee of Alexandria University Faculty of Dentistry (IRB No. 001056 – IORG 0008839) prior to any research-related activities.

2. Statement that all research activities involving human subjects will abide with the Declaration of Helsinki* and other ethical guidelines adopted by the Research Ethics Committee of Alexandria University Faculty of Dentistry.

3. The benefits to the participant and/or the community:
   - Removal of impacted mandibular third molars by using ER:YAG LASER

4. The risks to the participant:
   - Injury of inferior alveolar nerve
   - Mandible fracture

5. All measures will be taken to ensure the privacy and confidentiality of the participants.

---

* World Medical Association Declaration of Helsinki: ethical principles for medical research involving human subjects. JAMA 2013 27; 310(20):2191-4. doi: 10.10001/jama.

BUDGET ESTIMATE

No.	Items	Total price (L.E.)
1	24 panoramic x-rays (pre-operative)	6,720
2	24 CBCT (pre-operative)	14,400
3	24 panoramic x-rays (post-operative)	6,720
4	24 CBCT (post-operative)	14,400
5	Statistical analysis	1,500 L.E
6	Printing and Binding	4,000 L.E
7	Publication	5000 L.E
8	Computer writing	2,000 L.E
9	Other Costs	10,000
Total		64,740

DURATION OF STUDY

Estimated time: 11 Months

	By Months										
**Tasks**	**1**	**2**	**3**	**4**	**5**	**6**	**7**	**8**	**9**	**10**	**11**
**Sample selection/ patient rectuitment**	✓	✓									
**Clinical procedure Experimental procedure**	✓	✓									
**Follow up**	✓	✓	✓	✓	✓						
**Data management and statistical analysis**						✓					
**Writing Thesis/manuscript**						✓	✓	✓			
**Thesis submission**								✓	✓		

ANTICIPATED PROBLEMS

The main problem facing the study will be:

- Availability of patients having mesioangular mandibular third molars
- Follow up
- Maintenance.

PUBLICATION POLICY

Findings will be disseminated through peer-reviewed publication. The order of names will be:

- Karim Anwer Ahmed Nazmy
- Prof. Dr. Ahmed Adel Abdelhakim
- Prof. Josep Arnabat Dominguez
- Dr. Riham Moustafa Eldibany

REFERENCES

1. Farish SE, Bouloux GF. General technique of third molar removal. Oral Maxillofac Surg Clin North Am 2007;19:23-43, v-vi.
2. Rupprecht S, Tangermann K, Kessler P, Neukam FW, Wiltfang J. Er:YAG laser osteotomy directed by sensor controlled systems. J Craniomaxillofac Surg 2003;31:337-42.
3. Ge J, Yang C, Zheng JW, He DM, Zheng LY, Hu YK. Four osteotomy methods with piezosurgery to remove complicated mandibular third molars: a retrospective study. J Oral Maxillofac Surg 2014;72:2126-33.
4. Rashad A, Sadr-Eshkevari P, Heiland M, Smeets R, Prochnow N, Hoffmann E, et al. Practitioner experience with sonic osteotomy compared to bur and ultrasonic saw: a pilot in vitro study. Int J Oral Maxillofac Surg 2015;44:203-8.
5. Frank C, Gotter A. Are Laser Dental Procedures Better Than Traditional Treatments? 2019. Available at: https://www.healthline.com/health/laser-dentistry.
6. Ishikawa I, Aoki A, Takasaki AA. Clinical application of erbium:YAG laser in periodontology. J Int Acad Periodontol 2008;10:22-30.
7. Kreisler M, Kohnen W, Marinello C, Götz H, Duschner H, Jansen B, et al. Bactericidal effect of the Er:YAG laser on dental implant surfaces: an in vitro study. J Periodontol 2002;73:1292-8.

8. Maiti N, Sharma P, Jadon SS, Qadri F, Pamidi V, Ganvir SR, et al. Efficiency of Laser versus Bur in Impacted Mandibular Third Molar Surgery: An Original Research. J Pharm Bioallied Sci 2021;13:S1501-s5.
9. Rosner, B. Fundamentals of biostatistics. Nelson Education. 2015.
10. Universität Düsseldorf. G*Power.2019. Retrieved from http://www.gpower.hhu.de/.
11. Delgado DA, Lambert BS, Boutris N, McCulloch PC, Robbins AB, Moreno MR, et al. Validation of Digital Visual Analog Scale Pain Scoring With a Traditional Paper-based Visual Analog Scale in Adults. J Am Acad Orthop Surg Glob Res Rev 2018;2:e088.

Alexandria University
Faculty of Dentistry
Department of Pediatric Dentistry and
Dental Public Health

EFFICACY OF ER:YAG LASER IN REMOVAL OF IMPACTED MANDIBULAR THIRD MOLARS
(A RANDOMIZED CONTROLLED CLINICAL TRIAL)

Name: Karim Anwar Ahmad Nazmy Habs Elhaw

Summary Statement

Sample size was estimated based on assuming 95% confidence level and 80% study power. The mean mouth opening after 7 days was 33.45 ±2.32 mm for the conventional extraction technique and 30.85 ±1.96 mm for the laser group. [1] Based on difference between two independent means, a sample of 12 patients per group is required yielding effect size of 1.211. Total sample size= number per group × number of groups= 12 × 2= 24 patients.

Software

Sample size was based on Rosner's method [2] calculated by G*Power 3.0.10. [3]

References

1. Maiti N, Sharma P, Jadon SS, Qadri F, Pamidi VRCB, Ganvir SR, Tiwari RVC. Efficiency of Laser versus Bur in Impacted Mandibular Third Molar Surgery: An Original Research. *J Pharm Bioallied Sci.* 2021;13(Suppl 2):S1501-S1505.
2. Rosner, B. Fundamentals of biostatistics. *Nelson Education*. 2015.
3. Universität Düsseldorf. G*Power.2019. Retrieved from http://www.gpower.hhu.de/

Hams Abdelrahman; B.D.S, M.Sc

Hams Abdelrahman

Assistant lecturer of Dental Public Health
Alexandria University
Wednesday, July 3, 2024

Maha El Tantawi; B.D.S, M.Sc, Ph.D

Maha Tanta.

Professor of Dental Public Health
Alexandria University
Wednesday, July 3, 2024

Faculty of Dentistry, Alexandria University, Champolion st., Azarita, Alexandria 21521, Egypt
Tel: (203) 4868066/ 4869690 ext: 221-Fax: (203) 4868286

جمهورية مصر العربية
وزارة التعليم العالي
جامعة الإسكندرية

Egyptian University Libraries Consortium
EULC

نموذج رقم (6) المكتبة الرقمية لجامعة الاسكندرية

إفادة فحص موضوع الخطة البحثية

تفيد وحدة المكتبة الرقمية بجامعة الإسكندرية بأن: الباحث/ **كريم انور احمد نظمى حبس الحو** والمقيد على درجة/ **ماجستير** بقسم/ **ماجستير تطبيقات الليزر الإكلينيكى فى طب الأسنان** بكلية / **طب الاسنان** بأنه قد تم فحص موضوع الخطة البحثية المقدمة منه للتسجيل على درجة / **ماجستير** وعنوانها

كفاءة الليزر المائى في إزالة ضروس العقل المدفونة بالفك السفلي (تجربة إكلينيكية عشوائية محكمة)

Efficacy of ER: YAG LASER in removal of impacted mandibular third molars (A Randomized Controlled Clinical trial)

"وبعد البحث في المستودع الرقمي للرسائل المجازة وقيد الدراسة ببوابة اتحاد مكتبات الجامعات المصرية، قد تبين أن:

الموضوع غير مسجل حتى تاريخه.

تحررت هذه الإفادة بناءً على طلب الباحث لتقديمها إلى من يهمه الأمر.

مدة صلاحية الإفادة 3 أشهر فقط من تاريخ التحرير

الموظف المختص **مراجع و رئيس القسم**

تحريراً في 4/ 12 /2024

جامعة الاسكندرية – وحدة المكتبة الرقمية
الموقع الإلكتروني: www.eulc.edu.eg
البريد الإلكتروني: eulc@alexu.edu.eg

إدارة المكتبة الرقمية: 163طريق الحرية – الشاطبي
مبنى إدارة شئون المكتبات – الدور الثاني
تليفون / فاكس: 034295867

جامعة الاسكندرية
ALEXANDRIA UNIVERSITY

كليـــة طب الاسنان
FACULTY OF DENISTRY
كليـــــه معتمـــدة

إستمارة توافق مقترح الخطة البحثية لرسائل الماجستير و الدكتوراة مع الخطة الإستراتيجية للعلوم و التكنولوجيا و الإبتكار ٢٠٣٠

أولا:

- المسار الثاني في المحور: ---
- المشروع / الدراسة :---
- مسلسل المشروع / الدراسة :---
- الجهه التي إقترحت المشروع :---
- كود المشروع / الدراسة :---
- عنوان مقترح الخطة البحثية (البروتوكول):---

ثانياً :

في حال عدم إدراج مقترح الخطة البحثية لرسائل الماجستير و الدكتوراه ضمن ال ٦٤٨ مشروعاً دراسة في ثلاث عشر محورا للمسار الثاني لإستراتيجية العلوم و التكنولوجيا و الإبتكار (٢٠٣٠) تستوفي هذة الإستمارة مع الإشارة بوضوح أن مقترح الخطة البحثية لرسالة الماجستير و الدكتوراه ،لم يدرج في المشروعات و الدراسات للمحاور المختلفه بالمسار الثاني، مع إقتراح أن يضاف مشروع أو دراسة جديدة مع تحديد المحور الذى تندرج تحته مقترح الخطة البحثية (البروتوكول) و ذلك لرفع الأمر إلي وزارة التعليم العالي و البحث العلمي بالتوصية بإدراجها ضمن الدراسات و المشروعات لمحاور السمار الثاني مع تحديد الجهة المستفيدة .

المشرفون : التوقيع

الطالب : كريم انور احمد نظمي — كريم انور احمد

الإسم : أ.د/ احمد عادل عبد الحكيم — احمد

الإسم : أ.د/ ريهام الديباني — الديباني

الإسم : Prof. Dr./ Josep Arnabat

الإسم:

يعتمد رئيس مجلس القسم

الإسم : أ.د/ احمد عادل عبد الحكيم

التوقيع : احمد

جامعة الاسكندرية
ALEXANDRIA
UNIVERSITY

كليـة طب الاسنان
FACULTY OF DENISTRY
كليـــة معتمـــدة

IRB NO: 00010556 – IORG 0008839
https://ohrp.cit.nih.gov/search/search.aspx

تعهد من الباحث للجنة أخلاقيات البحوث الطبية

اسم الباحث: **كريم أنور أحمد نظمى**

اسم البحث باللغة العربية: **كفاءة الليزر المائى في إزالة ضروس العقل المدفونة بالفك السفلي (تجربة إكلينيكية عشوائية محكمة)**

اسم البحث باللغة الإنجليزية: **Efficacy of ER: YAG LASER in removal of impacted mandibular third molars (A Randomized Controlled Clinical trial)**

القسم العلمي: **ماجستير تطبيقات الليزر الإكلينيكى فى طب الأسنان**

عنوان الباحث: ٢٧٨ طريق الجيش، ستانلى، الإسكندرية

رقم الهاتف: ٠١١١١٦٨٧١١١

البريد الإلكتروني: dr.karim.nour@gmail.com

أتعهد أنا الموقع أدناه بمسئوليتي الكاملة عن استيفاء نموذج الموافقة المستنيرة المرفق بخطة البحث لكل مشارك قبل الخضوع للبحث، مع الاحتفاظ بأصل الموافقة المستنيرة وتسليم المشارك نسخة موقعة، ومن حق اللجنة الاطلاع عليها في أي وقت.

كما أؤكد أنه تم منح المشارك فرصة لطرح أسئلة حول الدراسة، وتم الإجابة على جميع الأسئلة التي طرحها المشارك بشكل صحيح وبقدر المستطاع، وأؤكد أن أي فرد لم يُجبر على إعطاء الموافقة، وأن الموافقة تم منحها طواعيه وبدون إكراه.

توقيع الباحث: كريم أنور أحمد نظمى

توقيع المشرف الرئيسي:

التاريخ:

الموافقة المستنيرة للاشتراك في دراسة علمية (يتبع ٢،١)

اسم الباحث: كريم أنور أحمد نظمى

القسم: ماجستير تطبيقات الليزر الإكلينيكى فى طب الأسنان

عنوان الدراسة: كفاءة الليزر المائى في إزالة ضروس العقل المدفونة بالفك السفلي (تجربة إكلينيكية عشوائية محكمة)

الهدف من الدراسة: تقييم مضاعفات ما بعد الجراحة بعد إزالة الأضراس الثالثة المنطمرة في الفك السفلي باستخدام Er:YAG LASER ونظام الحفر اليدوي التقليدي.

عدد الاشخاص المشاركين: ٤

خطوات الدراسة: تجربة إكلينيكية عشوائية محكمة

فترة مشاركتي بالدراسة: سنتين

فوائد الدراسة للمشارك وللمجتمع: إزالة ضروس العقل المدفونة بالفك السفلي باستخدام كفاءة الليزر المائى

أخطار الدراسة المحتملة: توفر المرضى الذين يعانون من الأضراس الثالثة الفكية المتوسطة، المتابعة والصيانة.

جامعة الإسكندرية
ALEXANDRIA
UNIVERSITY
كليـة طب الاسنان
FACULTY OF DENISTRY
كليـــــة معتمــــد ة

IRB NO: 00010556 – IORG 0008839
https://ohrp.cit.nih.gov/search/search.aspx

الموافقة المستنيرة للاشتراك في دراسة علمية (تابع ١)

سرية المعلومات:

سوف يتم حفظ المعلومات المتعلقة بك في سرية تامة ولن يتم التعريف بك في أي تقرير أو نشر لنتائج البحث.

وسائل علاج أي اصابة ربما تحدث من الدراسة:

سوف يقوم الباحث باتخاذ جميع الوسائل لمنع اي اصابة أو ضرر أو أي عراض جانبية من الممكن ان يحدث نتيجة لهذه الدراسة، ولكن إذا حدثت أي اصابة غير متوقعة نتيجة اشتراكك بالدراسة سوف تتلقي العلاج اللازم بالكلية؛ ولا يوجد أي تعويض مادي عن أي إصابة قد تنتج من هذه الدراسة.

تكاليف الدراسة: لا يوجد.

لن تتلقي اي اموال للاشتراك بالدراسة

ولكن يمكن أن تتلقى مقابلا رمزيا كبدل انتقال أو تعويض عن يوم عمل.

حقوقك كمشارك في الدراسة:

الاشتراك تطوعي ولك الحق في ان تستمر او تنسحب من الدراسة في أي وقت دون تبعات عليك.

للاستفسار عن الدراسة أو عند حدوث اي مشكلة او شكوى غير متوقعة او شعرت بأي اشياء غير طبيعية او معتادة يمكنك الاتصال برئيس لجنة أخلاقيات البحث العلمي عن طريق مكتب شئون الدراسات العليا بكلية طب الأسنان جامعة الإسكندرية.

إقرار الباحث:

لقد قمت بشرح الدراسة بالتفصيل والهدف منها وطريقة الدراسة والمخاطر والفوائد المتعلقة بالدراسة مع الاجابة عن أي أسئلة أثارها المشارك وسوف ألتزم بخطوات الدراسة كاملة وبالمعايير الاخلاقية والقانونية للبحث العلمي بالكلية، وفي حال تغيير خطوات البحث سيكون ذلك لمصلحة المريض وبإخطار مسبق للمريض، وللمشاركين الذين لا يحسنون القراءة والكتابة تم قراءة الموافقة المستنيرة على المشارك في حضور شاهد من اختيار المشارك.

كلية طب الأسنان جامعة الإسكندرية
شارع شامبليون الأزاريطه الإسكندرية – مصر. ت: /٤٨٦٨٠٦٦/٤٨٦٩٦٩٠(٢٠٣) فاكس ٤٨٦٨٢٨٦ (٢٠٣)

الموافقة المستنيرة للاشتراك في دراسة علمية (تابع٢)

موافقة المريض:

أنا الموقع ادناه قد شرح لي هدف وخطوات الدراسة، وقد استوعبت الفوائد والمخاطر المتعلقة بالدراسة وتم التأكيد على سرية معلوماتي الخاصة، وتم اخطاري باحتمال تغيير خطوات البحث عند الضرورة و لمصلحتي، وقد أخذت نسخة من هذه الموافقة، وقد تم اعطائي الفرصة للسؤال والاستفسار قبل التوقيع ، وقيل لي أن من حقي الاستفسار في اي وقت لاحق، وأنا أتطوع للمشاركة في هذه الدراسة و لي الحق في الانسحاب في اي وقت دون تبعات علي، وأوافق علي التعاون مع الباحث و إخباره مباشرة بأي مشاكل غير متوقعة يمكن ان تحدث لي أثناء اشتراكي في الدراسة.

اسم المشارك/ولي الأمر/ الممثل القانوني:

توقيع (ختم أو بصمة) المشارك/ولي الأمر/ الممثل القانوني:

توقيع الشاهد:
(عند عدم قدرة المشارك/ولي الأمر على القراءة والكتابة)

توقيع الباحث: ط/ إسراء أنور أحمد نظمي

التاريخ:

ETHICAL REVIEW REPORT

**Serial Number**	
**Research Title**	**Efficacy of ER: YAG LASER in removal of impacted mandibular third molars (A Randomized Controlled Clinical trial)**
**Researchers' names**	**Karim Anwer Ahmed Nazmy**<br>**Prof. Dr. Ahmed Adel Abdelhakim**<br>**Prof. Dr. Riham Eldibany**
**Submitted date**	
**Research Type**	☐ MBA ☐ MS ☒ MD ☐ Ph.D. ☐ MD-Ph.D. ☐ Research ☐ Project
**Reviewer report**	☐ Accepted after correction ☐ Not accepted
**Reasons**	

Signature of Reviewer [signature] **Signature date:**

Signature of Rapporteur [signature] **Signature date:**

ETHICS COMMITTEE CERTIFICATE OF EXEMPTION

**Serial Number**	
**Research Title**	**Efficacy of ER: YAG LASER in removal of impacted mandibular third molars (A Randomized Controlled Clinical trial)**
**Researchers' names**	**Karim Anwer Ahmed Nazmy**<br>**Prof. Dr. Ahmed Adel Abdelhakim**<br>**Prof. Dr. Riham Eldibany**
**Submitted date**	
**Research Type**	☐ MBA<br>☐ MS<br>☒ MD<br>☐ Ph.D.<br>☐ MD-Ph.D.<br>☐ Research<br>☐ Project

The application for ethical exemption for the research mentioned above was reviewed by the ethics committee of the faculty of .... on 00 - 00 - 2023 in compliance with the Alexandria University Ethics code and National regulation. **Ethical Exemption has been granted.**

Please be advised that the Ethics committee needs to be informed should any part of the research methodology change.

Reviewer's signature [signature] **Signature date:**

Rapporteur's signature [signature] **Signature date:**

جمهورية مصر العربية
وزارة التعليم العالي
جامعة الإسكندرية

نموذج رقم (14) المكتبة الرقمية لجامعة الاسكندرية

إفادة إيداع الخطة البحثية

تفيد وحدة المكتبة الرقمة بجامعة الاسكندرية بأن الباحث كريم انور احمد نظمى حبس الحر

بقسم ماجستير الإكلينيكى لتطبيقات الليزر فى طب الأسنان بكلية طب الاسنان

بأنه قام بإيداع الخطة البحثية المقدمة منه للتسجيل على درجة ماجستير

وعـــنـــوانـــهـــا

Efficacy of ER: YAG LASER in removal of impacted mandibular third molars (A Randomized Controlled Clinical trial)

كفاءة الليزر المائى في إزالة ضروس العقل المدفونة بالفك السفلي (تجربة إكلينيكية عشوائية محكمة)

الرقم الببليوجرافي ببوابة اتحاد مكتبات الجامعات المصرية : 9253263 BibID

تحررت هذه الافادة بناءً على طلب الباحث لتقديمها إلى من يهمه الامر.

مدة صلاحية الافادة 3 أشهر فقط من تاريخ التحرير

الموظف المختص

رئيس القسم

تحريراً فى: 2024-12-04

جامعة الاسكندرية – وحدة المكتبة الرقمية
الموقع الإلكتروني: www.eulc.edu.eg
البريد الإلكتروني: eulc@alexu.edu.eg

إدارة المكتبة الرقمية: 163طريق الحرية – الشاطبي
مبنى إدارة شئون المكتبات – الدور الثاني
تليفون / فاكس: 034295867
