# Supplementary material for: Efficacy of Er:YAG laser in removal of impacted mandibular third molars (a randomized controlled clinical trial)
Source: BMC Oral Health. 2026 Jun 5;26:1060. doi: 10.1186/s12903-026-08790-w (PMC13270853; doi:10.1186/s12903-026-08790-w)
Supplement: Supplementary file 3 — Supplementary Material 3. [file 12903_2026_8790_MOESM3_ESM.docx]

**Table S2. Pre-specified outcomes as approved prior to patient enrollment**

| **Outcome Type** | **Outcome** | **Measurement Method** | **Time Points** |
| --- | --- | --- | --- |
| Primary | Bone density | CBCT grayscale  (VOI-based) | Immediate, 3 months |
| Secondary | Pain | VAS (0-10) | Day 2, Day 7 |
| Secondary | Edema | Facial linear measurements | Pre-op, Day 2, Day 7 |
| Secondary | Trismus | Interincisal distance (mm) | Pre-op, Day 2, Day 7 |
| Secondary | Operation time | Minutes | Intraoperative |
