# Supplementary figures and images for: Efficacy of Er:YAG laser in removal of impacted mandibular third molars (a randomized controlled clinical trial)
Source: BMC Oral Health. 2026 Jun 5;26:1060. doi: 10.1186/s12903-026-08790-w (PMC13270853; doi:10.1186/s12903-026-08790-w)

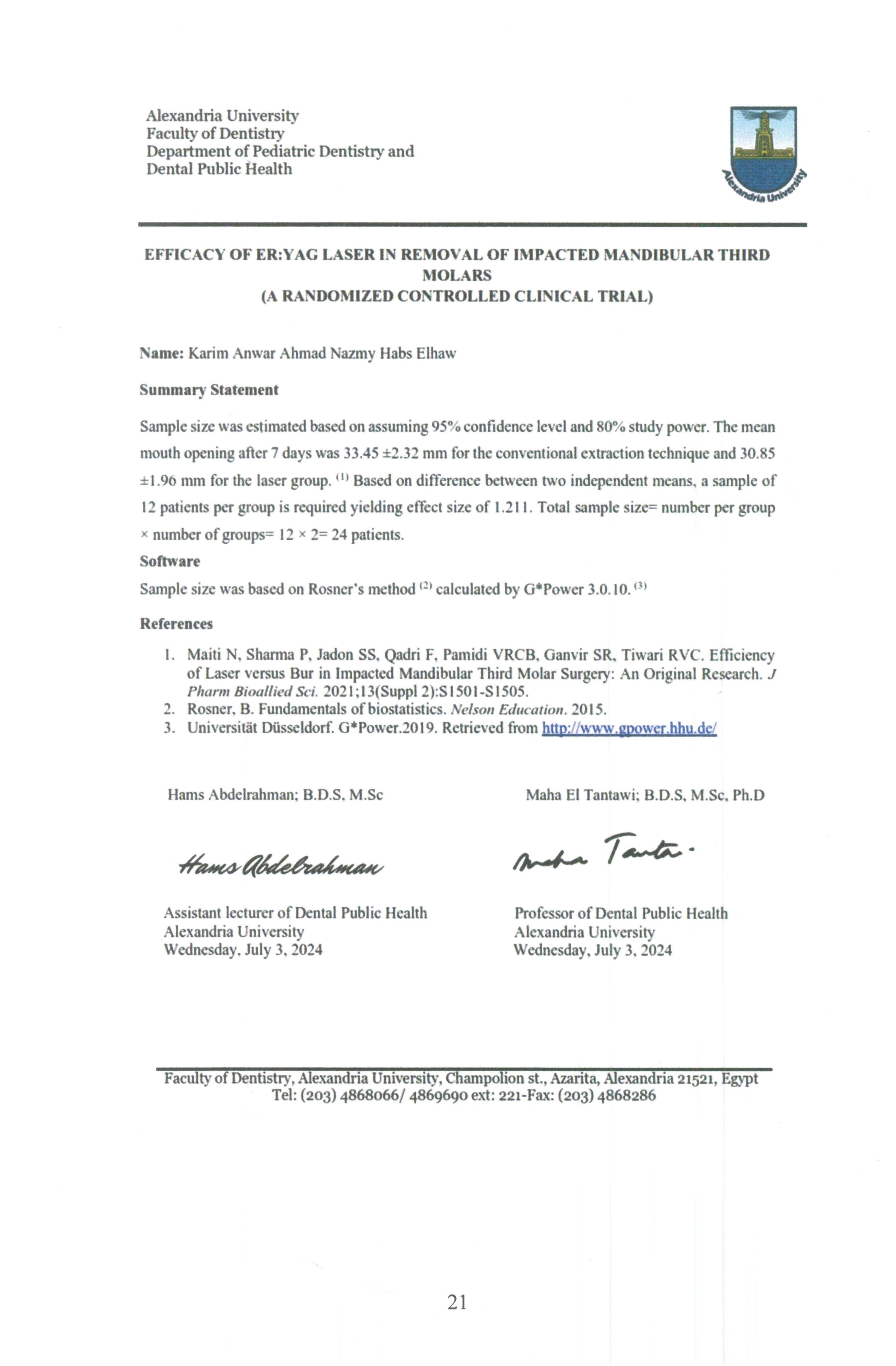

Supplement: Supplementary file 4 — Supplementary Material 4. [file 12903_2026_8790_MOESM4_ESM.jpg]
